# Supplementary material for: All-polymer organic solar cells with nano-to-micron hierarchical morphology and large light receiving angle
Source: Nat Commun. 2023 Jul 12;14:4148. doi: 10.1038/s41467-023-39832-4 (PMC10338490; doi:10.1038/s41467-023-39832-4)
Supplement: Supplementary file 1 — Supplementary Information [file 41467_2023_39832_MOESM1_ESM.pdf]

Supplementary Information

All-polymer organic solar cells with nano-to-micron hierarchical morphology and large light receiving angle

Rui Zeng[1§], Lei Zhu[1§], Ming Zhang[1], Wenkai Zhong[1], Guanqing Zhou[1], Jiaxing Zhuang[1], Tianyu Hao[1], Zichun Zhou[1], Libo Zhou[1], Nicolai Hartmann[2], Xiaonan Xue[3], Hao Jing[3], Fei Han[4], Yiming Bai[4], Hongbo Wu[5], Zheng Tang[5], Yecheng Zou[6], Haiming Zhu[7], Chun-Chao Chen[8], Yongming Zhang[1], Feng Liu[1]*

[1]School of Chemistry and Chemical Engineering, Frontiers Science Center for Transformative Molecules, In-situ Center for Physical Science, and Center of Hydrogen Science Shanghai Jiao Tong University, Shanghai 200240, China.

[2]Attocube systems AG, Eglfinger Weg 2, Haar 85540, Germany.

[3]Shanghai OPV Solar New Energy Technology Co., Ltd., Shanghai 201210, China.

[4]State Key Laboratory of Alternate Electrical Power System with Renewable Energy Sources, North China Electric Power University, Beijing 102206, China.

[5]Center for Advanced Low-dimension Materials, State Key Laboratory for Modification of Chemical Fibers and Polymer Materials, College of Materials Science and Engineering Donghua University, Shanghai 201620, China

[6]State Key Laboratory of Fluorinated Functional Membrane Materials and Dongyue Future Hydrogen Energy Materials Company, Zibo City, Shandong 256401, China.

[7]Department of Chemistry, Zhejiang University, Hangzhou, Zhejiang 310027, China.

[8]School of Materials Science and Engineering, Shanghai Jiao Tong University, Shanghai 200240, China.

§These authors contributed equally: Rui Zeng, Lei Zhu.

*e-mail: fengliu82@sjtu.edu.cn

Supplementary Methods

Energy level measurement of cyclic voltammetry (CV). The electrochemical behavior of the materials was investigated using CV with a standard three-electrode electrochemical cell in a 0.1 M Bu_4NPF_6 solution in CH_3CN at room temperature under an atmosphere of nitrogen with a scanning rate of 0.1 V/S. A Pt plate working electrode, a Pt wire counter electrode, and an Ag/AgCl (0.01 M in CH_3CN) reference electrode were used. The experiments were calibrated with the standard ferrocene/ferrocenium (Fc) redox system and assumption that the energy level of Fc is 4.37 eV below vacuum.

In site thickness variation curve measurement. In-situ thickness change was obtained using the FILMETRICS F20-EXR optical profilometer in continuous measurement setting.

Atomic force microscopy-based infrared spectroscopy (tapping AFM-IR). For tapping AFM-IR measurements, an IR-neaSCOPE (neaspec, attocube systems AG) was used to map IR absorption via detecting the mechanical response of an AFM cantilever upon sample stimulation by pulsed IR light. In the instrument, a pulsed, tunable QCL laser is focused onto a PtIr coated AFM probe via a parabolic mirror. while the microscope is operated in intermittent contact mode. The readout of the optically induced changes in the sample is performed by providing bimodal excitation of the cantilever and monitoring the response at the second cantilever Eigen-mode, while the first Eigen-mode is used for AFM topography and feedback. Such an active bimodal technique enables reliable tracking of the cantilever resonance, which in turn suppresses mechanically induced artefacts in the AFM-IR image contrasts. [1]

Transient absorption spectroscopy (TAS). For femtosecond transient absorption spectroscopy, the fundamental output from Yb:KGW laser (1030 nm, 220 fs Gaussian fit, 100 kHz, Light Conversion Ltd) was separated to two light beam. One was introduced to NOPA (ORPHEUS-N, Light Conversion Ltd) to produce a certain wavelength for pump beam (here we use 550 and 750 nm, 30 fs pulse duration), the

other was focused onto a YAG plate to generate white light continuum as probe beam. The pump and probe overlapped on the sample at a small angle less than 10°. The transmitted probe light from sample was collected by a linear CCD array.

The exciton annihilation method employs TAS to measure exciton lifetimes as a function of excitation density.[2] The exciton diffusion length, $L_D = \sqrt{D\tau}$. τ is the lifetime of single-excited for neat PY-IT films.

The series of fluence dependent decays are globally fit to a rate equation accounting for bimolecular (exciton annihilation) and monomolecular decay pathways, assuming that annihilation destroys both excitons;

$$\frac{\mathrm{d}n(t)}{\mathrm{d}t} = -kn(t) - \frac{1}{2}\gamma n^2(t) \tag{1}$$

which has the following solution:

$$n(t) = \frac{n(0)e^{(-kt)}}{1 + \frac{\gamma}{2k} n(0)[1 - e^{(-kt)}]} \tag{2}$$

where $n(t)$ is the singlet exciton density as a function of time after the laser excitation, k is the monomolecular decay rate and γ is the singlet-singlet bimolecular exciton annihilation rate. Here the intrinsic monomolecular decay constant, k, is a fixed value, extracted from the dilute PY-IT film where diffusion and annihilation does not occur. The global fits account for absolute excitation densities, which are ascertained from the TA signal after accounting for the fluence. The bimolecular rate constant is then used to determine the exciton diffusion coefficient

$$D = \frac{\gamma}{8\pi R} \tag{3}$$

where D is the diffusion constant and R is the effective interaction or annihilation radius of singlet excitons which is the separation at which the annihilation occurs.

Paracrystalline disorder factor (*g-factor*) on π-π stacking. The scherrer expression for weakly disordered system gives the coherence length that paracrystalline feature persists. And considering the interplanar distance *d*, *g*-factor could be estimated, defined by:[3, 4]

$$g = \frac{1}{2\pi}\sqrt{\Delta_q \times d} \tag{4}$$

Δ_q is the full width at half the maximum of a diffraction peak, defined as

$$\Delta_q = \frac{2\pi}{\mathrm{CCL}} \tag{5}$$

Combining the above two equations, we arrive at

$$g = \sqrt{\frac{d}{2\pi \times \mathrm{CCL}}} \tag{6}$$

Transient photovoltage (TPV) and photocurrent (TPC) measurement. The lifetime of carriers can be measured by the transient photovoltage measurement. The background illumination was provided by a normal LED light source, and pulsed light was provided by arbitrary wave generator. The photovoltage traces were registered by the oscilloscope. The photocurrent traces were registered with the resistance of 50 Ω, switching open-circuit mode to short-circuit mode. The integrated TPC signal provides a measure of the total charge generated by the laser pulse (ΔQ). Empirically, the differential capacitance values are found to follow the exponential dependence on the open-circuit voltage given by

$$C = \frac{\Delta Q}{\Delta V} = C_0 exp(\gamma V_{OC}) + D \tag{7}$$

So, the charge-carrier density as a function of V_{OC} is given by treating the device as a parallel-plate capacitor and integrating with respect to voltage, as

$$n = \frac{1}{Aed}\int_{-\infty}^{Voc} C_0 exp(\gamma V) dV \tag{8}$$

where A is the active layer area, and d is the active layer thickness. Then, the recombination rate coefficient can be determined, which is defined by

$$k(n) = \frac{1}{\tau(n)n} \tag{9}$$

The τ as a function of *n* follows an approximated exponential law of

$$\tau = \tau_0 (\frac{n}{n_0})^{\lambda} \tag{10}$$

suggesting that the non-geminate recombination is the dominant loss mechanism as the parameter of λ approaching 1. [5] The recombination order R (R=λ+1) was calculated by the exponential factor λ (λ equal to the slope of lg τ vs. lg *n*), R is expected to be 2 in

strictly bimolecular recombination conditions, and a higher value of R (R>2) measures the defects and inherent energy disorder that cause carrier recombination.

J_{SC} and V_{OC} on the light intensity (P_{light}) dependence. The correlation between J_{SC} and light intensity (P_{in}) can be expressed as $J_{SC} \approx P_{in}^{\alpha}$, where α is close to 1 suggesting minimal bimolecular recombination. [6] The dependence of V_{OC} on light intensity is examined to further understand the recombination processes. Generally, with a slope s close to kT/q (where k is the Boltzmann constant, T is the temperature in Kelvin, q is the elementary charge), trap-assisted recombination should be negligible.

Space-charge-limited current (SCLC) measurements. The electron-only devices were fabricated with ITO/ZnO/Active layer/PNDIT-F3N/Ag structures and hole-only devices were fabricated with ITO/PEDOT:PSS/Active layer/MoOx/Ag structures. The SCLC mobility was calculated according to the Mott-Gurney square law:

$$J=9\varepsilon_r\varepsilon_0\mu V^2/8L^3 \tag{11}$$

where J is the current density, ε_r is the relative dielectric constant of the transport medium component, ε_0 is the vacuum permittivity, μ is the electron or hole mobility, V is the effective voltage, and L is the thickness of active layer. [7]

Impedance spectroscopy (IS). IS is employed to access the DOS information. [8] The chemical capacitors (C_μ^n) determined by IS reflect the capability of the photovoltaic device to accept or release additional charge carriers as the result of the shifting in E_{Fn}. As the E_{Fn} reflects the electron filling level. Using the zero-temperature approximation of Fermi function at occupancy >1%, DOS $g_n(E_{Fn})$ is obtained by extracting the C_μ^n following:

$$C_\mu^n = Lq^2 g_n(E_{Fn}) \tag{12}$$

where L is the thickness of the active layer, q is the elemental charge. [9, 10] By applying different illumination and corresponding the bias voltage, the E_{Fn} moves across the

electronic states, and the scatter plot of filling state as a function of voltage can be obtained.

Impedance measurements were carried out by illumination with a 1.5G illumination source (1000 W m^{-2}) using a Solar Simulator. Impedance spectra were measured for different light intensities by applying a small voltage perturbation (10 mV rms) at frequencies from 8 MHz to 50 Hz, for different bias voltages. To measure in open circuit voltage conditions, a bias voltage equals to V_{OC} at each light intensity was applied. These measurements were performed with LCR-IM3536 equipped with a frequency analyzer module, always at room temperature. The data was collected for 10 average. Recombination resistance and chemical capacitance were directly extracted from the low-frequency region.

EQE_{EL} measurement. The EQE_{EL} was recorded with an in-house-built system comprising a standard silicon photodiode 1010B, Keithley 2400 source meter (for supplying voltages and recording injected currents), and Keithley 6482 picoammeter (for measuring the emitted light intensity).

Pearson correlation analysis. In the correlation analysis, we used the Pearson correlation coefficient r. The Pearson correlation coefficient r between two variables X and Y is defined as

$$r = \frac{cov(X,Y)}{\sigma_X \sigma_Y} \tag{13}$$

where $cov(X, Y)$ represents covariance between X and Y, σ_X and σ_Y represent respective standard deviations.

Refractive-index (*n*) and extinction-coefficient (*k*) measurement. The wavelength dependent refractive-index (*n*) and extinction-coefficient (*k*) was measured with SENTECH SE 850 DUV ellipsometer by Ms Le Zhao of National Center for Nanoscience and Technology.

Power generation simulation during summer solstice in Shanghai. Here is the formula for calculating the zenith angle θ_Z of Shanghai on that day.

$$\omega = 15 \times (ST - 12) \tag{14}$$

$$\alpha_s = \arcsin\,(sin\varphi sin\delta + cos\varphi cos\delta cos\omega) \tag{15}$$

$$\theta_Z = 90° - \alpha_s \tag{16}$$

ST is apparent solar time; φ is local latitude; δ is sun declination angle; ω is solar hour angle; α_s is solar altitude; θ_Z is zenith angle.

The formula of power generation on function of PCE is as follow.

$$P(mW) = PCE \times 100\, mW/cm^2 \times 0.052cm^2 \tag{17}$$

The power generation of as-cast (P_1) and sequential processing (P_2) is

$$P_1(mW) = (15.08 \times 1 + 14.90 \times 2 + 14.45 \times 2 + 13.72 \times 2 + 12.74 \times 2 + 11.49 \times 2) \times 0.052 = 7.78$$

$$P_2(mW) = (18.12 \times 1 + 18.08 \times 2 + 18.05 \times 2 + 17.98 \times 2 + 17.63 \times 2 + 16.08 \times 2) \times 0.052 = 10.08$$

$$P_2/P_1 = 1.30$$

Thus, the ratio of power generation between as-cast and sequential processing devices is 1.30.

Supplementary Tables

Supplementary Table 1. Photovoltaic performance in variety DIB concentration and SVA duration under simulated AM 1.5G illumination.

DIB	SVA (min)	J_{SC} (mA cm^{-2})	V_{OC} (V)	FF (%)	PCE[a] (%)
25 mg ml^{-1}	3	25.14 (24.32±0.22)	0.941 (0.939±0.003)	73.34 (72.78±0.73)	17.35 (16.63±0.31)
	5	25.69 (25.27±0.17)	0.940 (0.937±0.002)	74.10 (73.58±0.61)	17.89 (17.41±0.26)
	7	25.48 (25.01±0.19)	0.941 (0.939±0.003)	72.49 (72.17±0.46)	17.38 (16.94±0.18)
30 mg ml^{-1}	3	25.40 (24.89±0.24)	0.944 (0.938±0.005)	73.85 (73.14±0.69)	17.71 (17.09±0.32)
	5	25.95 (25.55±0.18)	0.943 (0.939±0.002)	74.51 (74.13±0.50)	18.24 (17.79±0.22)
	7	25.73 (25.25±0.20)	0.937 (0.937±0.004)	73.06 (72.51±0.57)	17.62 (17.15±0.29)
35 mg ml^{-1}	3	24.70 (24.28±0.25)	0.941 (0.938±0.002)	72.79 (72.06±0.77)	16.92 (16.41±0.36)
	5	25.37 (24.94±0.21)	0.939 (0.938±0.003)	73.17 (72.79±0.49)	17.43 (17.02±0.24)
	7	25.27 (24.79±0.18)	0.940 (0.939±0.004)	71.76 (71.42±0.58)	17.04 (16.67±0.26)

[a]The average values are obtained from ~30 individual experimental results.

Supplementary Table 2. Crystallization analysis for all-PSC films (in-plane).

Lamellar ($q \approx 0.297$ Å^{-1})	Peak Area	*d*-spacing (Å)	CCL (Å)
As-cast	92.31±4.14	21.20	58.02
DIB	129.01±5.57	21.39	70.11
DIB/TA/SVA	133.36±5.96	21.14	73.82
DIB/SVA/TA	111.51±7.73	21.19	71.31
($q \approx 0.395$ Å^{-1})	Peak Area	*d*-spacing (Å)	CCL (Å)
As-cast	29.61±1.03	16.37	68.61
DIB	54.88±2.51	15.91	74.40
DIB/TA/SVA	57.94±2.20	15.88	76.59
DIB/SVA/TA	53.96±4.69	16.00	74.00

Note: The error of d-spacing and CCL both less than 0.01 Å, which is due to the statistical properties of the scattering technology itself.

Supplementary Table 3. Crystallization analysis for all-PSC films (out-of-plane).

π-π ($q \approx 1.640$ Å^{-1})	Peak Area	d-spacing (Å)	CCL (Å)
As-cast	1024.72±9.58	3.84	16.63
DIB	1047.16±10.22	3.84	18.29
DIB/TA/SVA	1162.85±9.76	3.84	19.27
DIB/SVA/TA	1028.03±12.64	3.85	18.16
Amorphous ($q \approx 1.279$ Å^{-1})	Peak Area	d_{ap} (Å)	
As-cast	140.81±4.52	4.95	
DIB	128.73±4.16	4.94	
DIB/TA/SVA	118.12±3.88	4.94	
DIB/SVA/TA	140.40±6.94	4.94	

Supplementary Table 4. Photovoltaic performance of as-cast and DIB/TA/SVA as function of light receiving angle under simulated AM 1.5G illumination based on PM6:PY-IT.

PM6:PY-IT	Angle (°)	J_{SC} (mA cm^{-2})	V_{OC} (V)	FF (%)	PCE (%)
As-cast	0	24.17 (23.68±0.24)	0.933 (0.932±0.001)	66.87 (66.31±0.55)	15.08 (14.64±0.27)
	10	23.53 (23.08±0.24)	0.928 (0.928±0.001)	67.05 (66.53±0.55)	14.64 (14.25±0.26)
	20	22.49 (21.98±0.26)	0.922 (0.921±0.002)	67.19 (66.65±0.57)	13.93 (13.49±0.28)
	30	21.39 (20.83±0.27)	0.916 (0.914±0.002)	67.28 (66.72±0.58)	13.18 (12.70±0.28)
	40	20.06 (18.49±0.30)	0.910 (0.908±0.002)	67.43 (66.84±0.60)	12.31 (11.23±0.25)
	50	18.05 (17.44±0.31)	0.903 (0.901±0.003)	67.62 (67.03±0.61)	11.02 (10.54±0.29)
DIB/TA/SVA	0	25.89 (25.52±0.22)	0.942 (0.941±0.001)	74.32 (73.88±0.54)	18.12 (17.75±0.29)
	10	25.80 (25.41±0.24)	0.939 (0.939±0.001)	74.45 (73.96±0.56)	18.03 (17.66±0.31)
	20	25.77 (25.34±0.25)	0.935 (0.934±0.001)	74.65 (74.14±0.56)	17.98 (17.56±0.30)
	30	25.69 (25.20±0.27)	0.932 (0.931±0.002)	74.68 (74.10±0.58)	17.87 (17.40±0.32)
	40	25.30 (24.78±0.27)	0.927 (0.925±0.002)	74.71 (74.14±0.59)	17.52 (17.01±0.32)
	50	24.29 (23.77±0.30)	0.923 (0.921±0.002)	74.78 (74.18±0.61)	16.76 (16.25±0.34)

[a]The average values are obtained from ~30 individual experimental results.

In order to reduce the impact of the edge shadow, the devices are measured without mask.

Supplementary Table 5. Photovoltaic performance of as-cast and DIB/TA/SVA as function of light receiving angle under simulated AM 1.5G illumination based on PM6:Y6.

PM6:Y6	Angle (°)	J_{SC} (mA cm^{-2})	V_{OC} (V)	FF (%)	PCE (%)
As-cast	0	25.16 (24.76±0.22)	0.867 (0.866±0.001)	70.31 (69.84±0.55)	15.33 (14.98±0.25)
	10	24.29 (23.92±0.23)	0.862 (0.860±0.001)	70.52 (69.98±0.55)	14.77 (14.40±0.26)
	20	23.38 (22.95±0.25)	0.858 (0.858±0.001)	70.74 (70.21±0.56)	14.19 (13.84±0.26)
	30	22.06 (21.63±0.28)	0.853 (0.851±0.001)	70.91 (70.38±0.58)	13.34 (12.95±0.27)
	40	20.32 (19.84±0.31)	0.848 (0.846±0.002)	71.24 (70.69±0.58)	12.27 (11.85±0.29)
	50	18.17 (17.60±0.35)	0.843 (0.842±0.002)	71.45 (70.86±0.60)	10.94 (10.50±0.30)
DIB/TA/SVA	0	26.29 (25.95±0.23)	0.857 (0.856±0.001)	75.80 (75.38±0.54)	17.08 (16.75±0.28)
	10	26.06 (25.64±0.25)	0.855 (0.855±0.001)	75.87 (75.47±0.55)	16.90 (16.58±0.29)
	20	25.92 (25.51±0.24)	0.853 (0.852±0.001)	75.96 (75.53±0.56)	16.79 (16.43±0.29)
	30	25.61 (25.12±0.29)	0.850 (0.848±0.001)	76.10 (75.65±0.58)	16.57 (16.12±0.32)
	40	25.16 (24.65±0.32)	0.848 (0.846±0.001)	76.26 (75.75±0.58)	16.27 (15.81±0.33)
	50	24.54 (23.97±0.34)	0.845 (0.844±0.001)	76.47 (75.92±0.59)	15.86 (15.38±0.34)

[a]The average values are obtained from ~30 individual experimental results.

In order to reduce the impact of the edge shadow, the devices are measured without mask.

Supplementary Table 6. Photovoltaic performance of as-cast and DIB/TA/SVA as function of light receiving angle under simulated AM 1.5G illumination based on D18:Y6.

D18:Y6	Angle (°)	J_{SC} (mA cm^{-2})	V_{OC} (V)	FF (%)	PCE (%)
As-cast	0	25.56 (24.98±0.24)	0.869 (0.869±0.001)	70.62 (70.06±0.62)	15.69 (15.22±0.28)
	10	24.62 (24.00±0.29)	0.866 (0.864±0.002)	70.71 (70.12±0.64)	15.08 (14.55±0.32)
	20	23.57 (22.75±0.33)	0.862 (0.860±0.001)	70.83 (70.21±0.64)	14.39 (13.74±0.32)
	30	22.46 (21.61±0.37)	0.856 (0.855±0.001)	71.02 (70.36±0.65)	13.65 (13.01±0.34)
	40	20.65 (19.73±0.44)	0.849 (0.847±0.002)	71.26 (70.54±0.67)	12.49 (11.79±0.39)
	50	18.52 (17.60±0.46)	0.842 (0.841±0.001)	71.54 (70.76±0.69)	11.16 (10.48±0.37)
DIB/TA/SVA	0	26.84 (26.32±0.26)	0.849 (0.847±0.001)	76.35 (75.86±0.59)	17.40 (16.97±0.30)
	10	26.68 (26.07±0.29)	0.846 (0.844±0.002)	76.42 (75.95±0.62)	17.25 (16.72±0.33)
	20	26.45 (25.69±0.32)	0.843 (0.841±0.001)	76.51 (76.10±0.64)	17.06 (16.46±0.33)
	30	26.02 (25.29±0.36)	0.839 (0.838±0.001)	76.78 (76.17±0.63)	16.76 (16.15±0.36)
	40	25.39 (24.50±0.41)	0.835 (0.834±0.002)	76.96 (76.31±0.66)	16.32 (15.61±0.39)
	50	24.64 (23.69±0.45)	0.830 (0.829±0.001)	77.19 (76.53±0.67)	15.79 (15.03±0.41)

[a]The average values are obtained from ~30 individual experimental results.

In order to reduce the impact of the edge shadow, the devices are measured without mask.

Supplementary Table 7. Photovoltaic performance of as-cast and DIB/TA/SVA as function of light receiving angle under simulated AM 1.5G illumination based on PM6:IT-4F.

PM6:IT-4F	Angle (°)	J_{SC} (mA cm^{-2})	V_{OC} (V)	FF (%)	PCE (%)
As-cast	0	20.19 (19.90±0.19)	0.860 (0.859±0.001)	72.40 (71.95±0.44)	12.57 (12.32±0.19)
	10	19.23 (18.90±0.20)	0.858 (0.859±0.001)	72.47 (72.00±0.46)	11.96 (11.71±0.20)
	20	18.36 (18.02±0.19)	0.856 (0.855±0.002)	72.55 (72.08±0.46)	11.40 (11.13±0.20)
	30	16.91 (16.54±0.22)	0.852 (0.850±0.001)	72.79 (72.29±0.47)	10.49 (10.17±0.20)
	40	15.38 (14.99±0.23)	0.846 (0.844±0.002)	73.02 (72.55±0.48)	9.50 (9.18±0.20)
	50	13.47 (13.03±0.25)	0.837 (0.836±0.002)	73.31 (72.78±0.50)	8.27 (7.93±0.21)
DIB/TA/SVA	0	21.17 (20.87±0.19)	0.863 (0.862±0.001)	74.96 (74.51±0.44)	13.69 (13.40±0.23)
	10	21.08 (20.77±0.19)	0.862 (0.860±0.001)	75.05 (74.59±0.44)	13.64 (13.34±0.23)
	20	20.96 (20.65±0.20)	0.861 (0.859±0.001)	75.11 (74.63±0.45)	13.55 (13.26±0.23)
	30	20.75 (20.42±0.21)	0.859 (0.859±0.001)	75.25 (74.77±0.45)	13.41 (13.12±0.24)
	40	20.38 (20.01±0.22)	0.854 (0.852±0.001)	75.42 (74.91±0.46)	13.13 (12.78±0.24)
	50	19.54 (19.15±0.23)	0.848 (0.847±0.001)	75.67 (74.21±0.47)	12.54 (12.05±0.22)

[a]The average values are obtained from ~30 individual experimental results.

In order to reduce the impact of the edge shadow, the devices are measured without mask.

Supplementary Table 8. Photovoltaic performance of as-cast and DIB/TA/SVA as function of light receiving angle under simulated AM 1.5G illumination based on PBDB-T:ITIC.

PBDB-T:ITIC	Angle (°)	J_{SC} (mA cm^{-2})	V_{OC} (V)	FF (%)	PCE (%)
As-cast	0	15.56 (15.08±0.28)	0.897 (0.895±0.002)	62.07 (61.45±0.61)	8.66 (8.29±0.24)
	10	14.94 (14.46±0.30)	0.893 (0.890±0.002)	62.14 (61.51±0.63)	8.29 (7.92±0.25)
	20	14.26 (13.90±0.33)	0.889 (0.887±0.001)	62.23 (61.57±0.64)	7.89 (7.60±0.25)
	30	13.07 (12.72±0.32)	0.883 (0.880±0.002)	62.41 (61.72±0.67)	7.20 (6.91±0.24)
	40	11.58 (11.17±0.36)	0.875 (0.873±0.003)	62.60 (61.88±0.69)	6.34 (6.03±0.25)
	50	9.62 (9.19±0.40)	0.866 (0.863±0.003)	62.92 (62.17±0.70)	5.24 (4.93±0.26)
DIB/TA/SVA	0	17.41 (17.09±0.27)	0.897 (0.895±0.001)	68.26 (67.65±0.60)	10.66 (10.34±0.25)
	10	17.14 (16.81±0.29)	0.895 (0.894±0.001)	68.34 (67.74±0.61)	10.48 (10.17±0.26)
	20	16.85 (16.50±0.32)	0.892 (0.890±0.002)	68.45 (67.82±0.63)	10.28 (9.95±0.27)
	30	16.38 (16.04±0.32)	0.886 (0.884±0.002)	68.63 (67.97±0.63)	9.96 (9.64±0.28)
	40	15.86 (15.47±0.35)	0.879 (0.876±0.002)	68.91 (68.23±0.66)	9.61 (9.24±0.30)
	50	15.21 (14.78±0.38)	0.871 (0.868±0.003)	69.24 (68.55±0.68)	9.17 (8.80±0.31)

[a]The average values are obtained from ~30 individual experimental results.

In order to reduce the impact of the edge shadow, the devices are measured without mask.

Supplementary Table 9. 2D volumn integrate values in different incident angle light.

	0°	10°	20°	30°	40°	50°
As-cast	$6.19*10^{36}$	$6.01*10^{36}$	$5.80*10^{36}$	$5.39*10^{36}$	$5.10*10^{36}$	$4.51*10^{36}$
DIB/TA/SVA	$7.12*10^{36}$	$7.07*10^{36}$	$7.03*10^{36}$	$6.99*10^{36}$	$6.89*10^{36}$	$6.71*10^{36}$

Supplementary Table 10. Changes of zenith angle during summer solstice in Shanghai.

ST (h)	ω (°)	φ (°)	δ (°)	α_s (°)	θ_Z (°)	Normalized θ_Z
7	-75	41	23.4	26.09	63.91	46.31
8	-60	41	23.4	37.36	52.64	35.04
9	-45	41	23.4	48.62	41.38	23.78
10	-30	41	23.4	59.36	30.64	13.04
11	-15	41	23.4	68.37	21.63	4.03
12	0	41	23.4	72.4	17.60	0
13	15	41	23.4	68.37	21.63	4.03
14	30	41	23.4	59.36	30.64	13.04
15	45	41	23.4	48.62	41.38	23.78
16	60	41	23.4	37.36	52.64	35.04
17	75	41	23.4	26.09	63.91	46.31

Normalized θ_Z gives the device an inclination of 17.6° so that it could be perpendicular to sunlight at noon.

Supplementary Table 11. The efficiency corresponding to the angle of incidence at different times.

ST (h)	corresponding θ_Z	PCE (%)	
		As-cast	DIB/TA/SVA
7	46.31	11.49	17.08
8	35.04	12.74	17.63
9	23.78	13.72	17.98
10	13.04	14.45	18.05
11	4.03	14.90	18.08
12	0	15.08	18.12
13	4.03	14.90	18.08
14	13.04	14.45	18.05
15	23.78	13.72	17.98
16	35.04	12.74	17.63
17	46.31	11.49	17.08

Supplementary Table 12. TAS parameters of neat PY-IT films in different conditions with varying excited fluence. The data was achieved through exponential fitting.

	D (cm^2 s^{-1})	τ (ps)	L_D (nm)
As-cast	4.72*10^{-2}	266.35±6.56	35.46
DIB	5.18*10^{-2}	274.77±6.87	37.72
DIB/TA/SVA	5.49*10^{-2}	289.63±6.95	39.87

Supplementary Table 13. TAS parameters of blend films in different conditions. The data was achieved through biexponential fitting.

	τ_1 (ps)	A_1	τ_2 (ps)	A_2	τ_p (ps)
As-cast	0.531±0.026	0.276±0.007	13.706±0.824	0.669±0.006	1498.7±21.67
DIB	0.446±0.021	0.359±0.007	12.071±0.610	0.592±0.006	1955.1±24.94
DIB/TA/SVA	0.416±0.023	0.424±0.008	11.365±0.546	0.542±0.011	2151.9±27.37

Supplementary Table 14. Parameters fitted in TPC/TPV and light intensity experiment. (n is charge density, τ_c is the charge lifetimes, α is the parameter about non-geminate recombination, s is the curve slope of voltage versus light intensity)

	λ	n ($\times10^{19}$ m^{-3})	τ_c (μs)	α	s (kT/q)
As-cast	1.34±0.008	5.31	1.19	0.935±0.001	1.188±0.051
DIB	1.31±0.004	7.64	1.33	0.936±0.002	1.129±0.037
DIB/TA/SVA	1.30±0.005	8.78	1.39	0.938±0.001	1.105±0.044
DIB/SVA/TA	1.45±0.013	1.45	0.84	0.931±0.005	1.493±0.081

Supplementary Table 15. Mobilities obtained from SCLC measurements.

	μ_e (cm^2 s^{-1} V^{-1})	μ_h (cm^2 s^{-1} V^{-1})	μ_e/μ_h
As-cast	(1.12±0.288)×10^{-3}	(3.01±0.352)×10^{-4}	3.72
DIB	(1.22±0.323)×10^{-3}	(3.52±0.339)×10^{-4}	3.47
DIB/TA/SVA	(1.27±0.274)×10^{-3}	(3.71±0.404)×10^{-4}	3.42
DIB/SVA/TA	(8.96±0.522)×10^{-4}	(2.04±0.597)×10^{-4}	4.39

Supplementary Table 16. DoS details for all-PSC in different condition.

	N_t (cm^{-3})	E_t (meV)
As-cast	(3.36±0.282)×10^{18}	139±0.9
DIB	(6.00±0.361)×10^{18}	118±1.4
DIB/TA/SVA	(1.28±0.190)×10^{19}	99±0.7
DIB/SVA/TA	(2.18±0.524)×10^{18}	162±2.5

Supplementary Figures

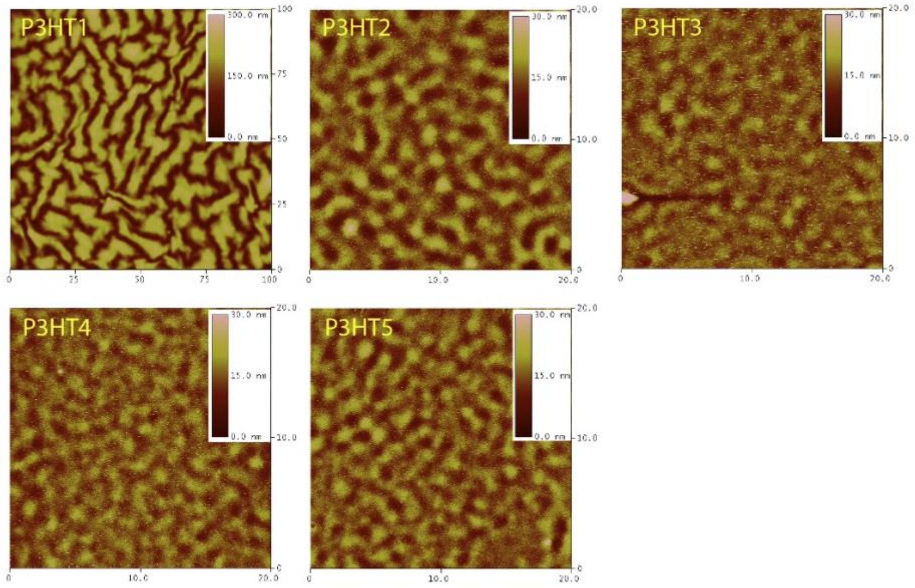

Supplementary Fig. 1 The previous experimental data about surface optical morphology based on P3HT:PCBM in our group.

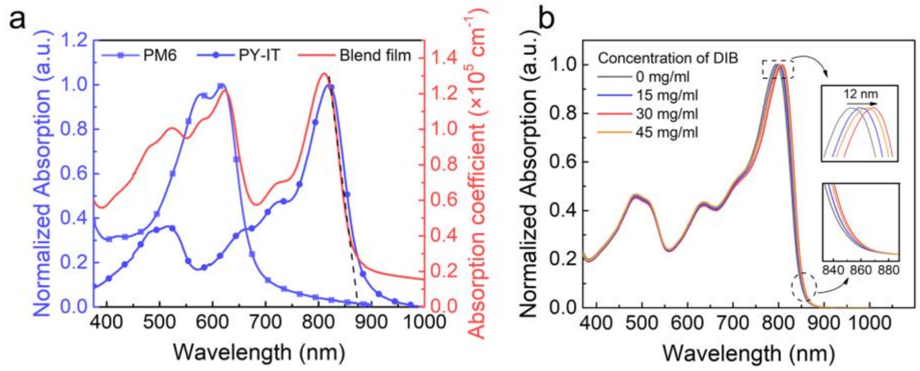

Supplementary Fig. 2 a, UV-vis absorption spectroscopy of pure and blend films, **b,** normalized absorption spectra of the neat PY-IT in different concentration of DIB.

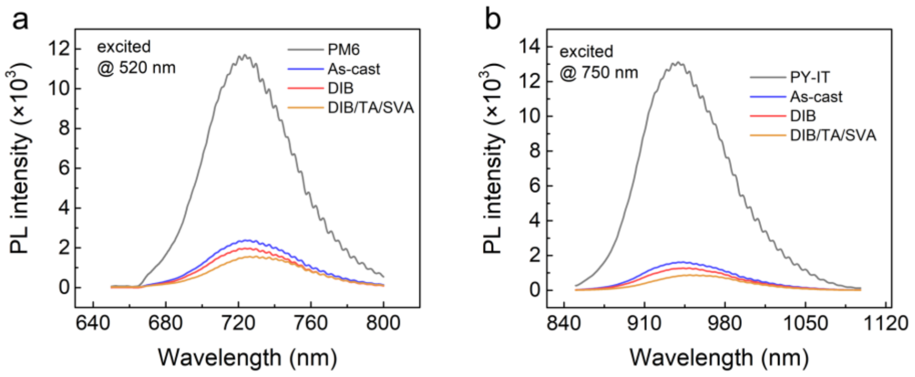

Supplementary Fig. 3 PL spectra of **a,** PM6 and blends in different conditions excited at 520 nm, **b,** PY-IT and blends in different conditions excited at 750 nm.

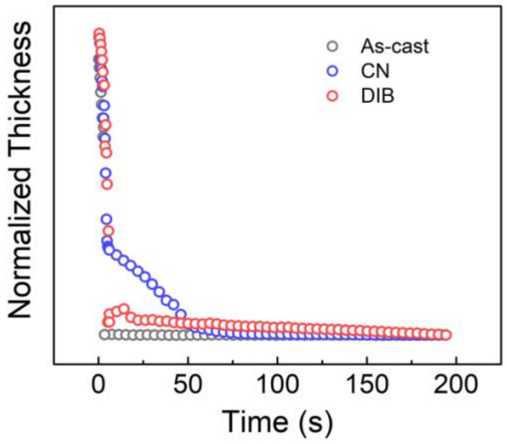

Supplementary Fig. 4 In site thickness variation curve of polymer films with as-cast, CN, DIB (in CF) in steady solvent evaporation process.

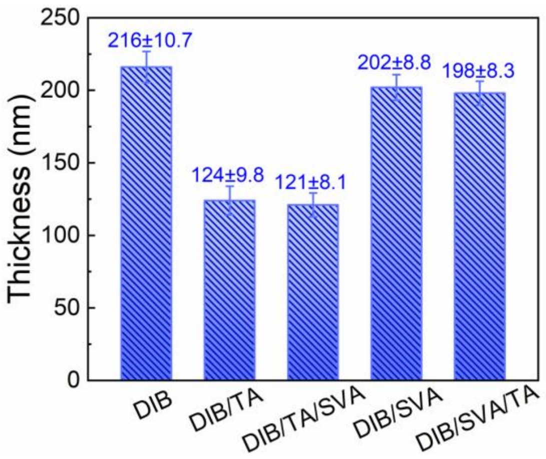

Supplementary Fig. 5 The film thickness in DIB, DIB/TA, DIB/TA/SVA, DIB/SVA, DIB/SVA/TA process. The values are obtained from around 10 experimental data.

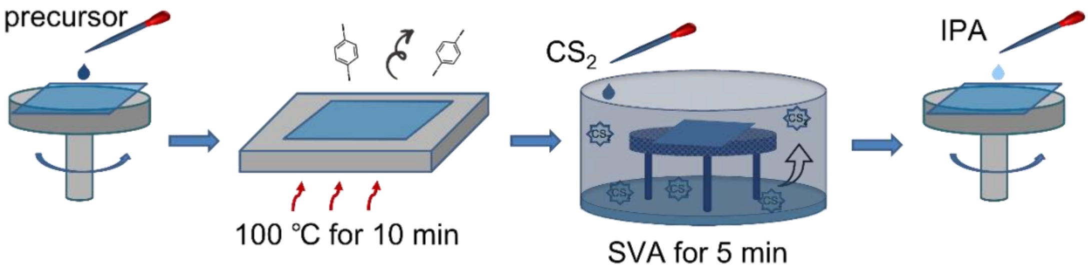

Supplementary Fig. 6 Schematic diagram of process routing for DIB/TA/SVA.

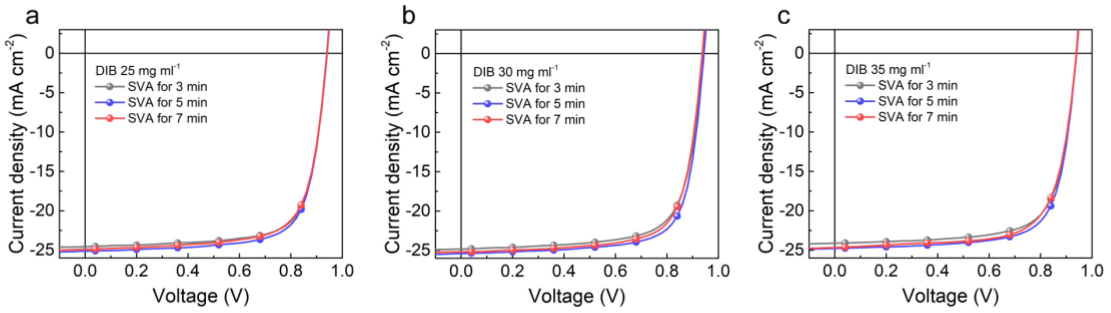

Supplementary Fig. 7 Device performance in **a,** 25 mg ml^{-1} DIB, **b,** 30 mg ml^{-1} DIB, **c,** 35 mg ml^{-1} DIB and SVA for 3/5/7 min.

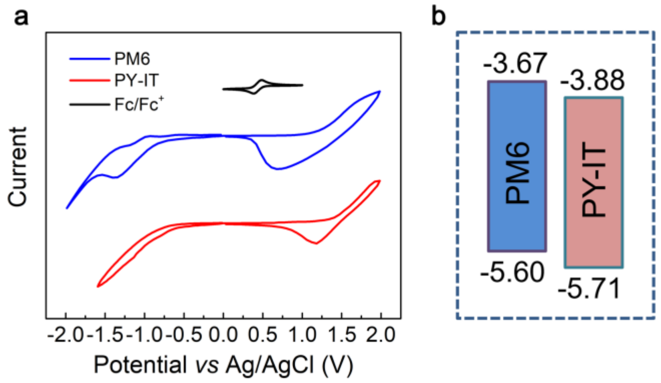

Supplementary Fig. 8 Energy level measurement of **a,** CV for PM6 and PY-IT neat films, and **b,** Energy level diagram (unit, eV).

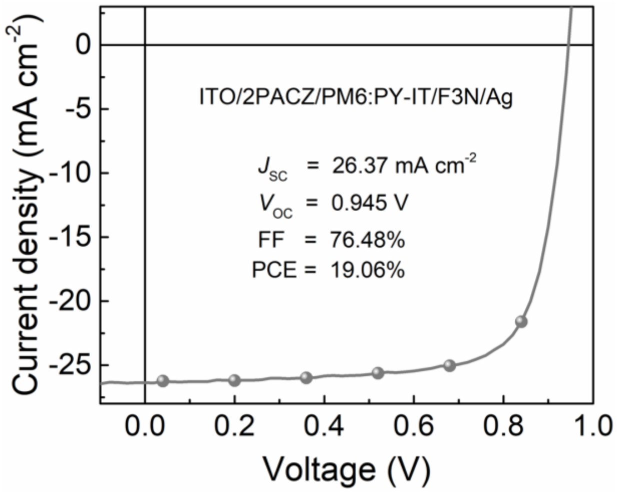

Supplementary Fig. 9 the efficiency of ITO/2PACZ/PM6:PY-IT/F3N/Ag in optimum condition.

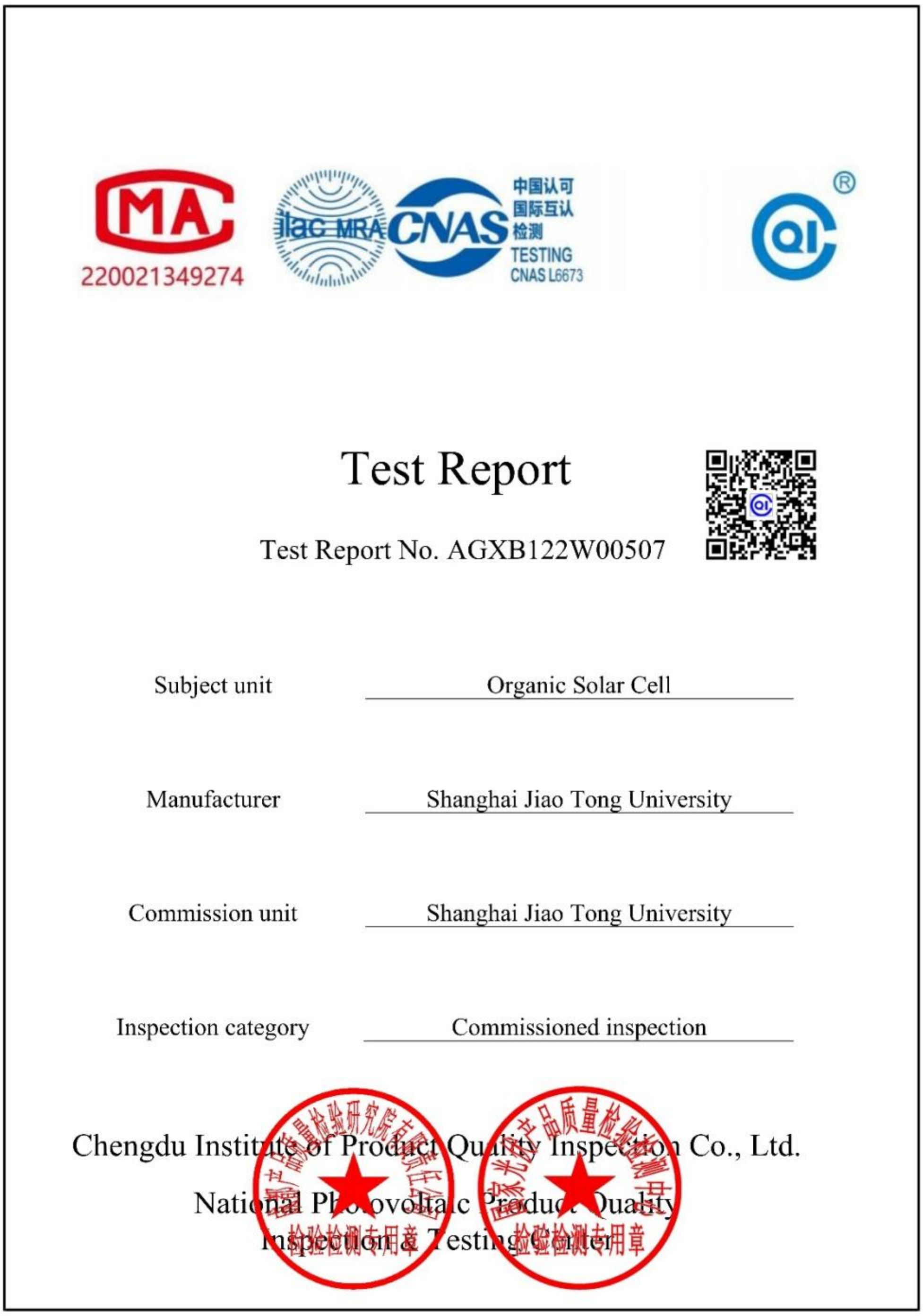

220021349274

Test Report

Test Report No. AGXB122W00507

Subject unit	Organic Solar Cell
Manufacturer	Shanghai Jiao Tong University
Commission unit	Shanghai Jiao Tong University
Inspection category	Commissioned inspection

Chengdu Institute of Product Quality Inspection Co., Ltd.

National Photovoltaic Product Quality

Inspection & Testing Center

Supplementary Fig. 10 The certification test report of the best performance by the Chengdu Institute of Product Quality Inspection Co., Ltd. National Photovoltaic Product Quality Inspection &Testing Center. (the cover of the report)

Chengdu Institute of Product Quality Inspection Co., Ltd.
National Photovoltaic Product Quality Inspection &Testing Center
TEST REPORT

Test Report No.AGXB122W00507 Page 1 of 3

Product Name	Organic Solar Cell	Trade Mark	/
Manufacture Date	16/11/2022	Model /Type	/
Sample No.	AGXB122W00507	Sample Grade	/
Sample Quantity	One piece	Sample State	Normal
Delivery Date	18/11/2022	Sample Delivered personnel	Feng Liu
Commission unit	Shanghai Jiao Tong University	Manufacturer	Shanghai Jiao Tong University
Commission unit address	No. 800 Dongchuan Road	Manufacturer Address	No. 800 Dongchuan Road
Commission unit Zip code	200240	Manufacturer Zip code	200240
Commission unit Tel.	17602152711	Manufacturer Tel.	17602152711
Center Address	No. 355, 2nd Tengfei Road, Southwest Airport Economic Development Zone, Chengdu, Sichuan, P. R. China.	Measurement Date	18/11/2022
Methods	IEC 60904-1:2006 Photovoltaic devices-Part 1: Measurement of Photovoltaic Current-Voltage Characteristics.		
Test conclusion	This column blank. (Special chapter for test report) Issue Date 22/11/2022		
Remarks	The mask area is provided by the Commission unit: 0.0313 cm^2; Original number of the sample:Z15-4.		
**Approved by** 夏庚培	**Reviewed by** [illegible]	**Measured by** [illegible]	

成都产品质量检验研究院有限责任公司 检验检测专用章

国家光伏产品质量检验检测中心 检验检测专用章

Supplementary Fig. 11 The basic information of the test sample, commission unit, and third-party agency. (page 1 of the report)

Chengdu Institute of Product Quality Inspection Co., Ltd.
National Photovoltaic Product Quality Inspection &Testing Center
TEST REPORT

Test Report No. AGXB122W00507 Page 2 of 3

Test Results:

No.	Test item(s)	Unit	Results
1	Current-voltage characteristics measurement	---	---
1.1	Open-circuit voltage, V_{OC}	V	0.945
1.2	Short-circuit current, I_{SC}	mA	0.822
1.3	Maximum-power, P_{max}	mW	0.582
1.4	Maximum-power voltage, $V_{p\text{-}max}$	V	0.791
1.5	Maximum-power current, $I_{p\text{-}max}$	mA	0.736
1.6	Fill factor, FF	%	74.95
1.7	Conversion efficiency, η	%	18.59

Current-voltage characteristics

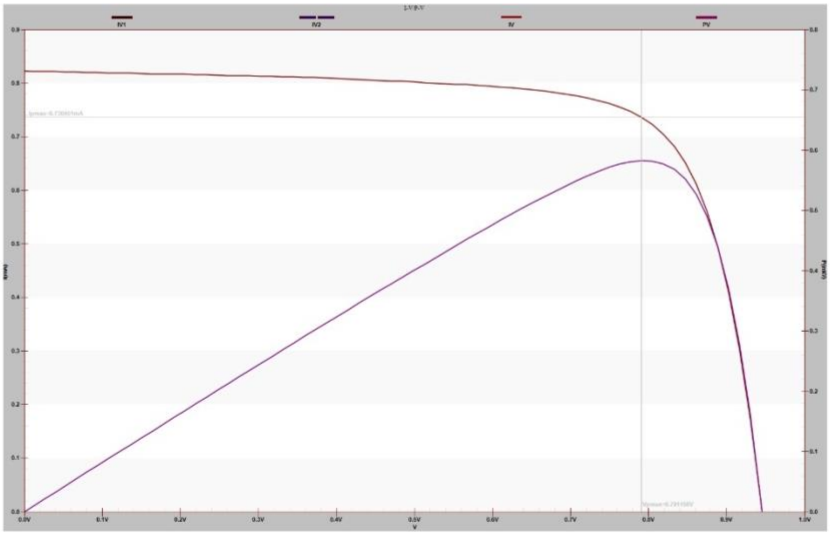

Remark: Sample was tested under the irradiation with a steady-state class calibrated AAA solar simulator (AM1.5-G 1000.0 W/m² basted on mono-Si reference cell) at 24±1 °C. Designated area defined by Position 4 on the left of thin metal aperture mask.

Supplementary Fig. 12 The specific performance parameters of the test sample. (page 2 of the report)

The mask

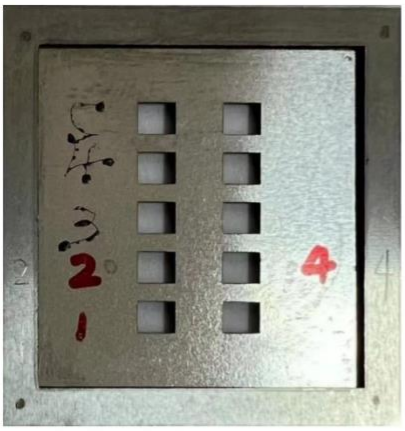

———Blank———

Supplementary Fig. 13 The mask used in the test. (page 3 of the report)

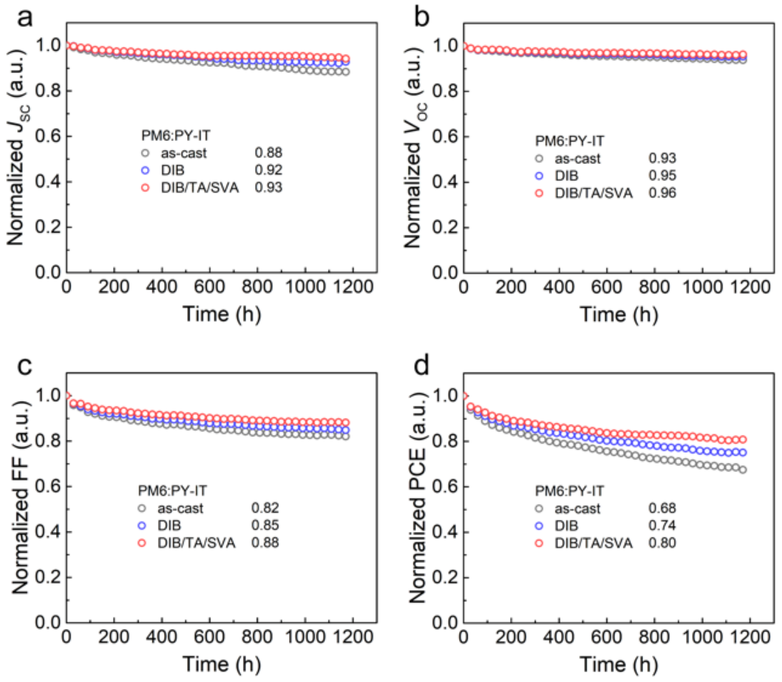

Supplementary Fig. 14 Continuous illumination stability of normalized **a,** J_{CS} **b,** V_{OC} **c,** FF **d,** PCE based on PM6:PY-IT in different conditions.

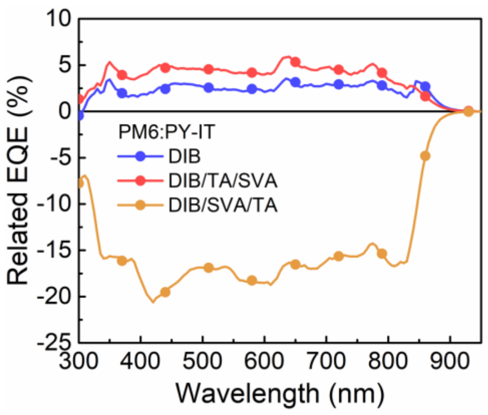

Supplementary Fig. 15 Relative EQE curves of PM6:PY-IT in different condition compared to as-cast device.

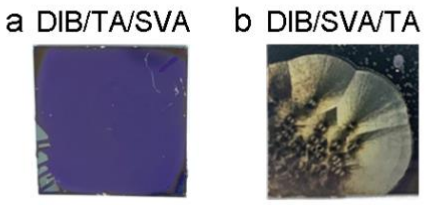

Supplementary Fig. 16 The BHJ in **a,** DIB/TA/SVA condition and **b,** DIB/SVA/TA condition prepared on silicon wafer.

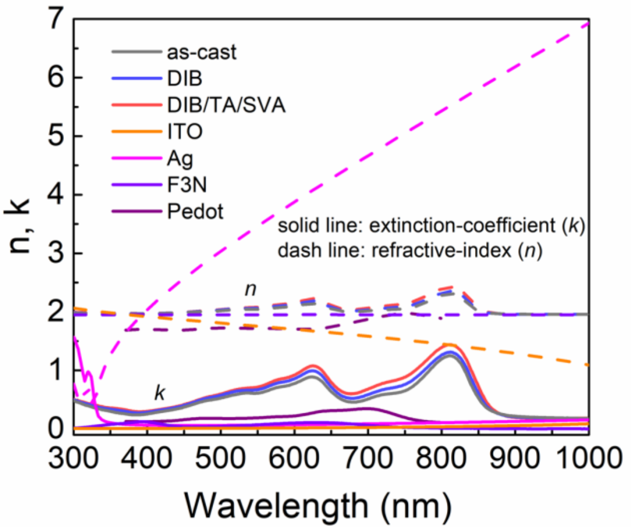

Supplementary Fig. 17 Refractive index (*n*) and extinction coefficient (*k*) of the involved layers for optical modeling.

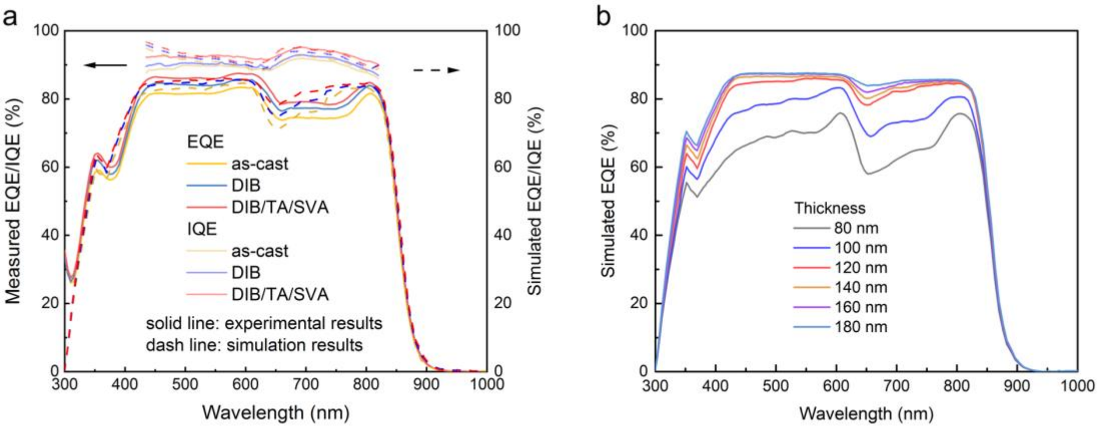

Supplementary Fig. 18 a, the measured EQE/IQE (solid line) vs simulated EQE/IQE (dash line), **b,** the simulated EQE as a function of junction thickness.

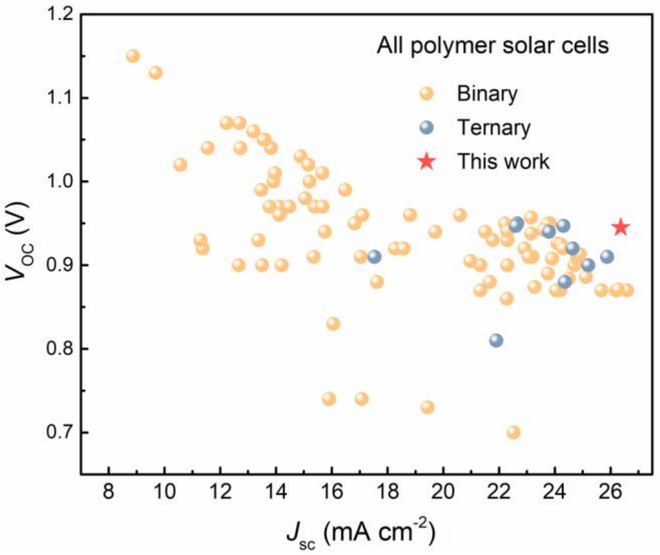

Supplementary Fig. 19 Plots of the V_{OC} versus J_{SC} for the efficient all-PSCs reported in the literature.

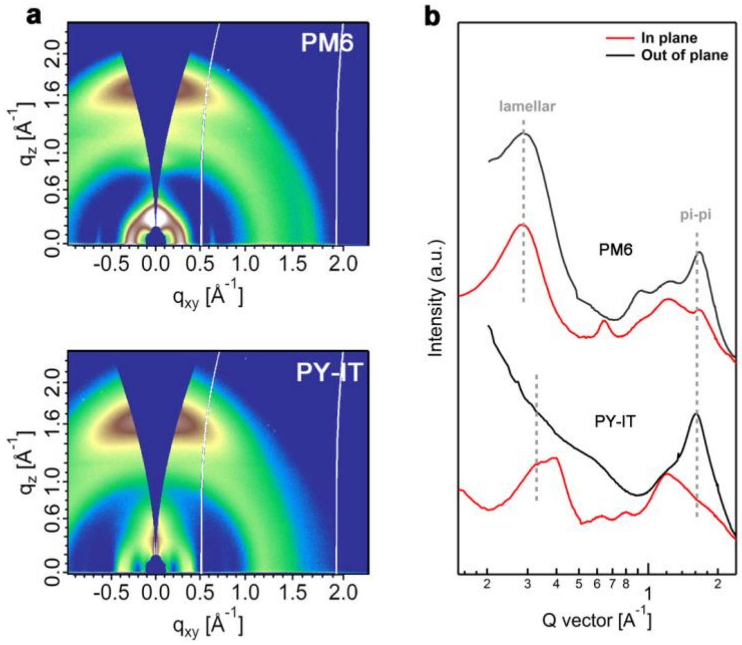

Supplementary Fig. 20 a, 2D GIWAXS patterns and **b,** line cut profiles for PM6 and PY-IT neat films.

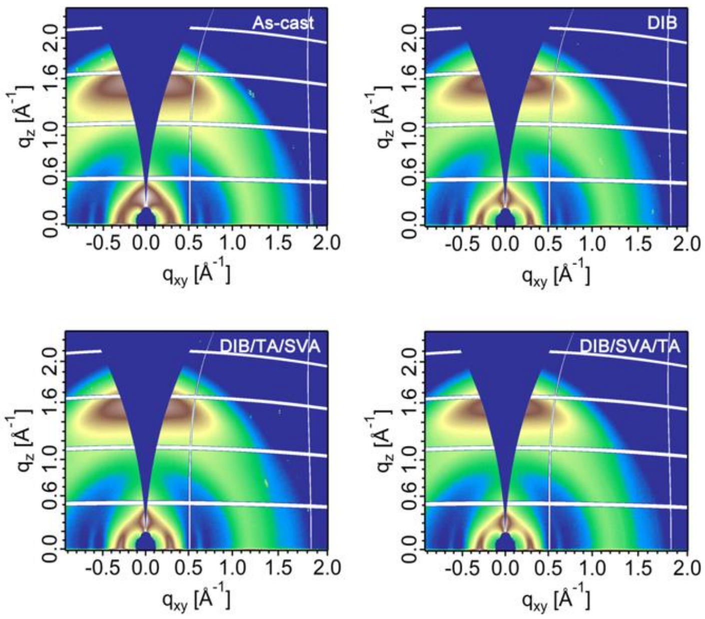

Supplementary Fig. 21 2D GIWAXS patterns for PM6:PY-IT blends in different conditions.

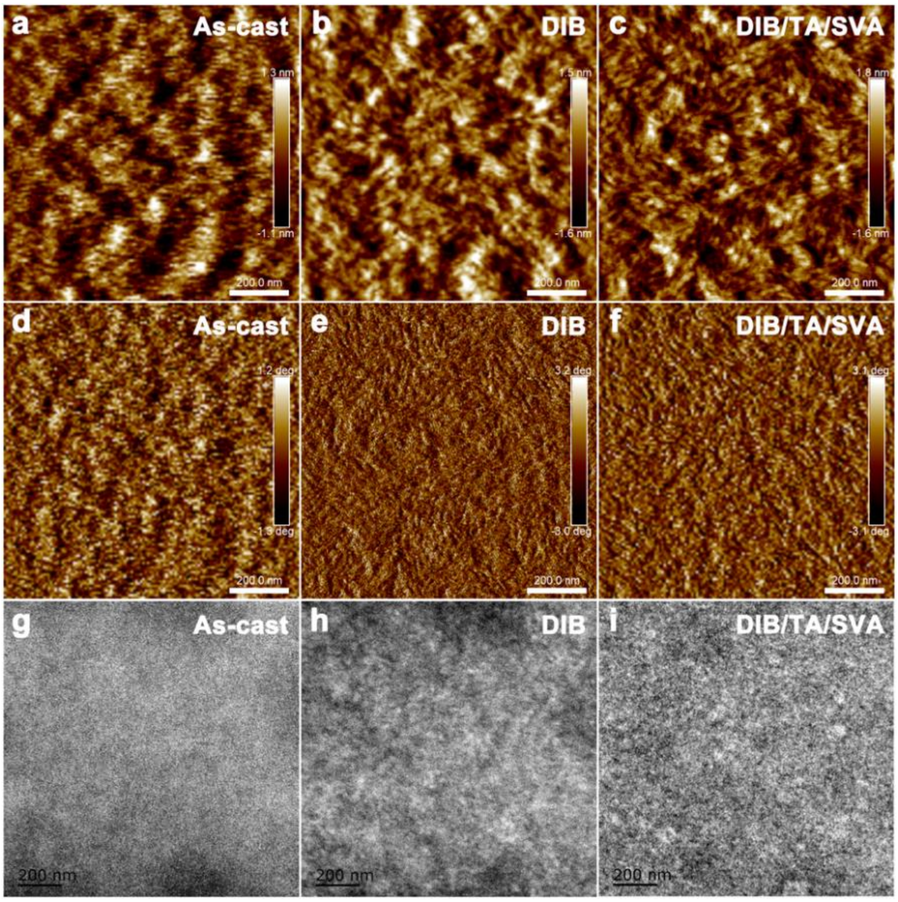

Supplementary Fig. 22 Morphology for PM6:PY-IT BHJ films of **a-c,** height images, **d-f,** phase images, and **g-i,** TEM images in different conditions.

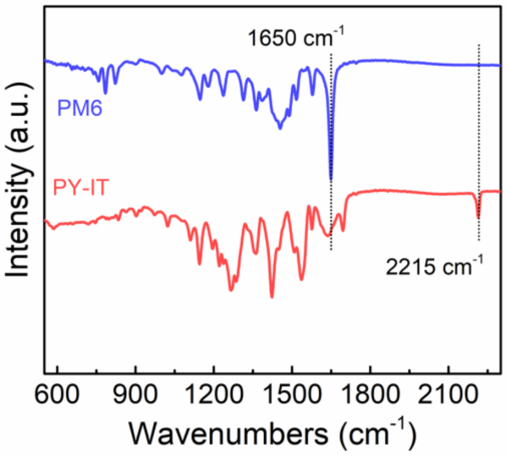

Supplementary Fig. 23 FTIR spectra of PM6 and PY-IT.

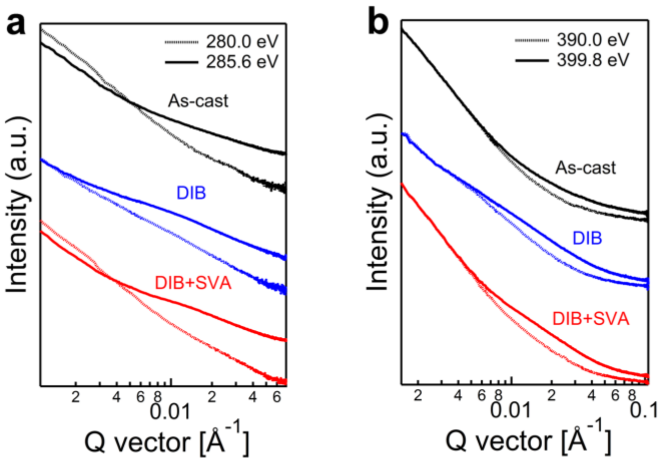

Supplementary Fig. 24 RSoXS measurement of **a,** CK-RSoXS profiles and **b,** NK-RSoXS profiles for blended films.

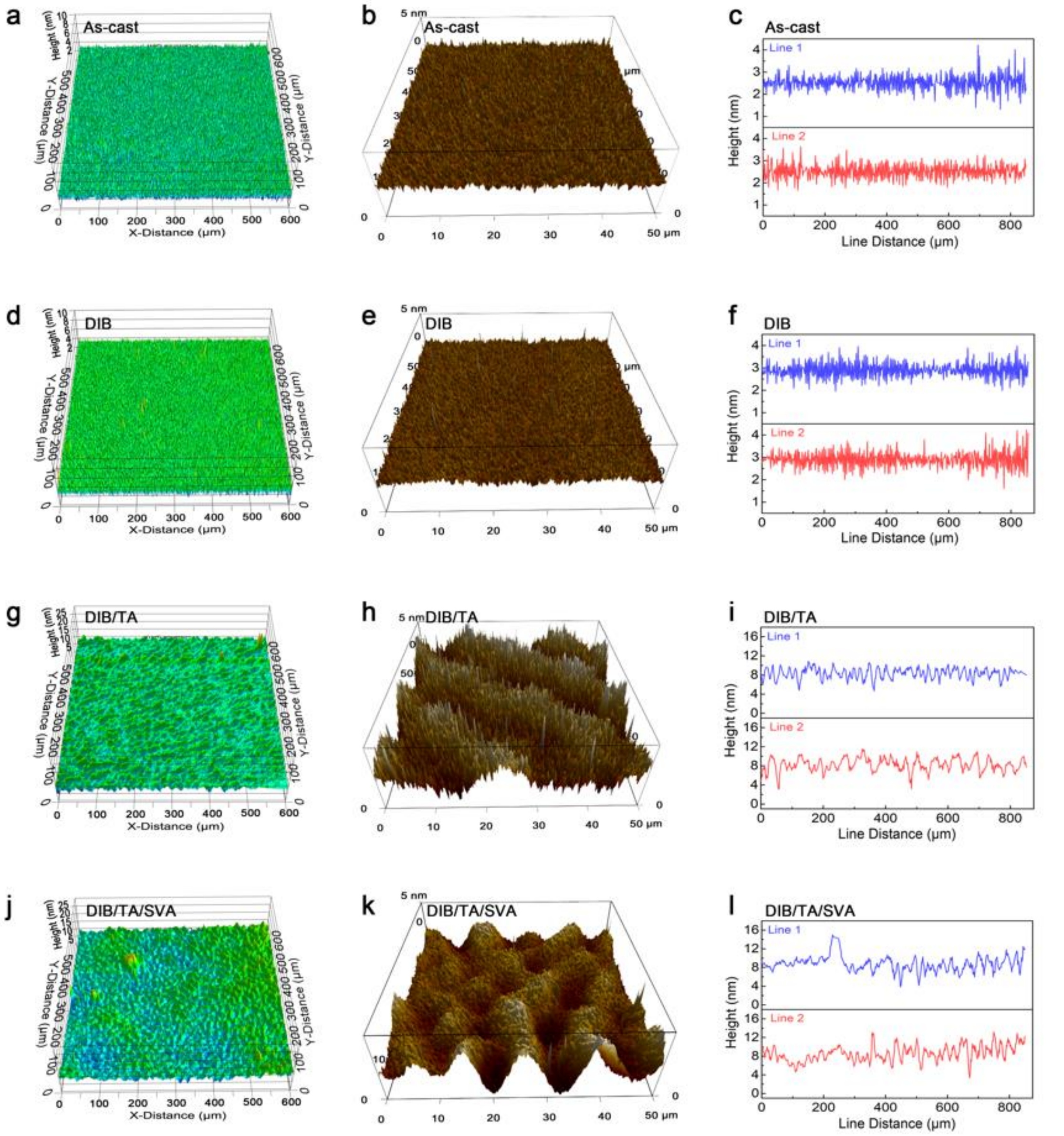

Supplementary Fig. 25 **a**, Surface profile, **b**, three-dimensional AFM, **c**, diagonal linecut of surface profile of as-cast. **d**, Surface profile, **e**, three-dimensional AFM, **f**, diagonal linecut of surface profile of DIB. **g**, Surface profile, **h**, three-dimensional AFM, **i**, diagonal linecut of surface profile of DIB/TA. **j**, Surface profile, **k**, three-dimensional AFM, **l**, diagonal linecut of surface profile of DIB/TA/SVA.

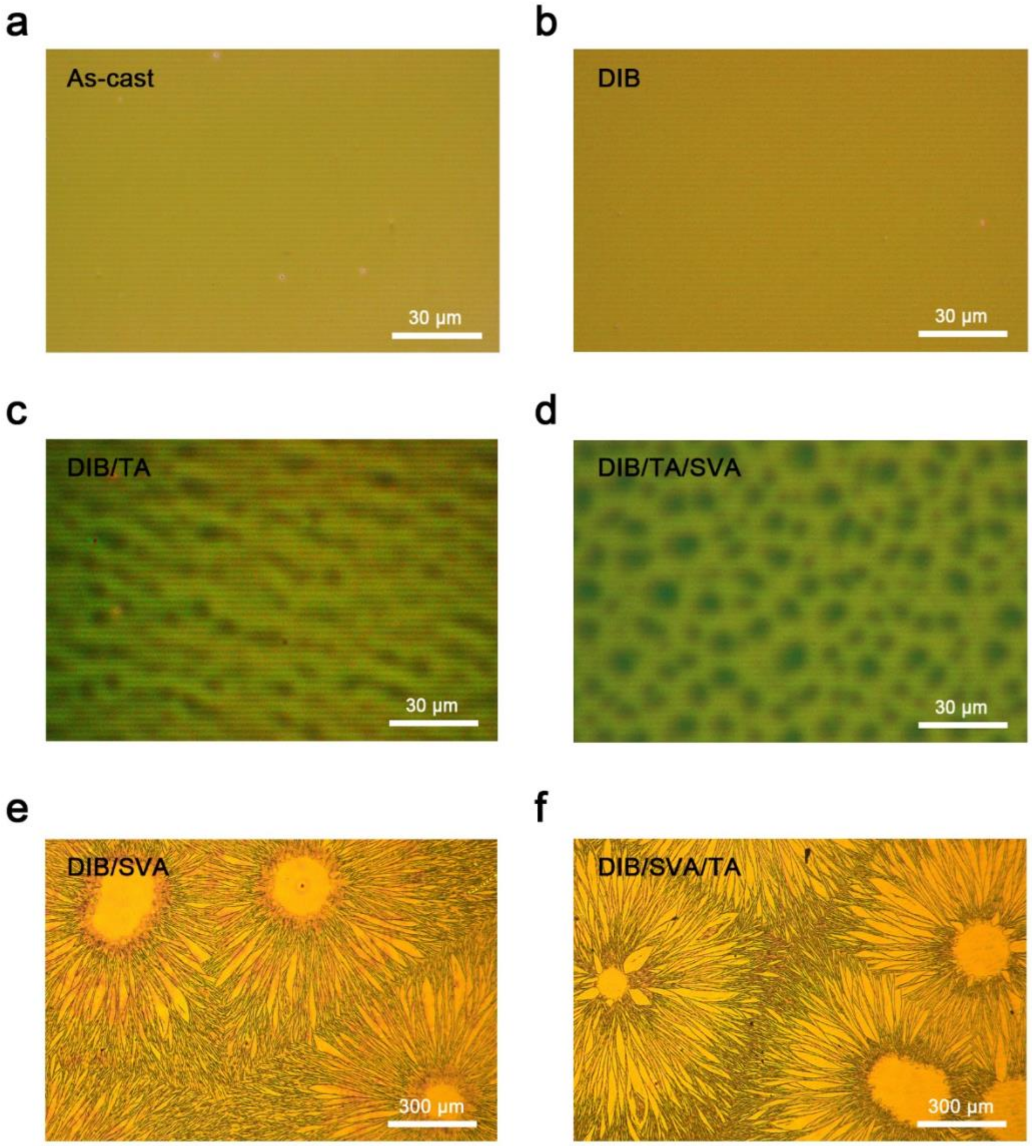

Supplementary Fig. 26 Optical microscope photograph of **a**, as-cast **b**, DIB **c**, DIB/TA **d**, DIB/TA/SVA **e**, DIB/SVA **f**, DIB/SVA/TA.

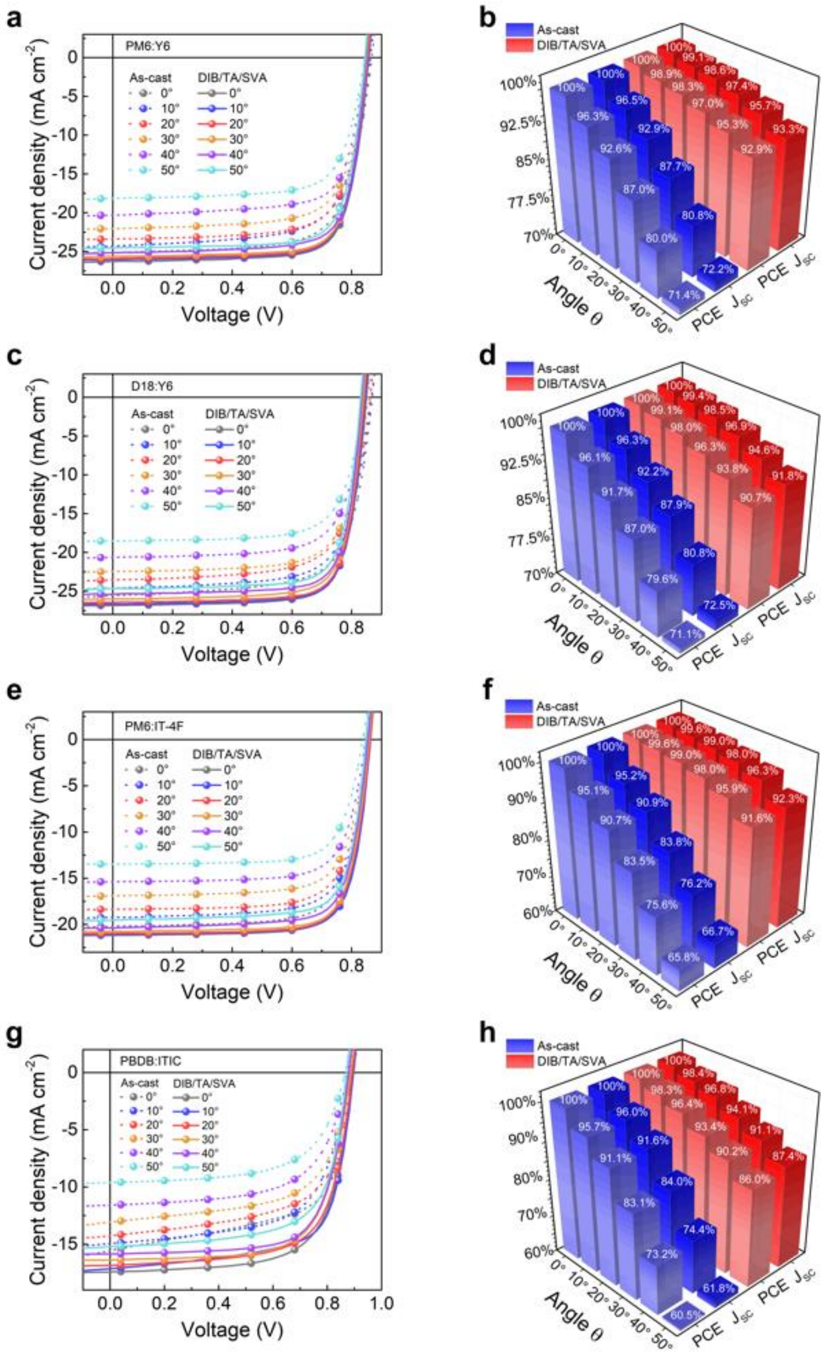

Supplementary Fig. 27 *J*-*V* curves and decay histogram of as-cast and sequential processing as function of light receiving angle for **a, b,** PM6:Y6, **c, d,** D18:Y6, **e, f,** PM6:IT-4F, **g, h,** PBDB-T:ITIC.

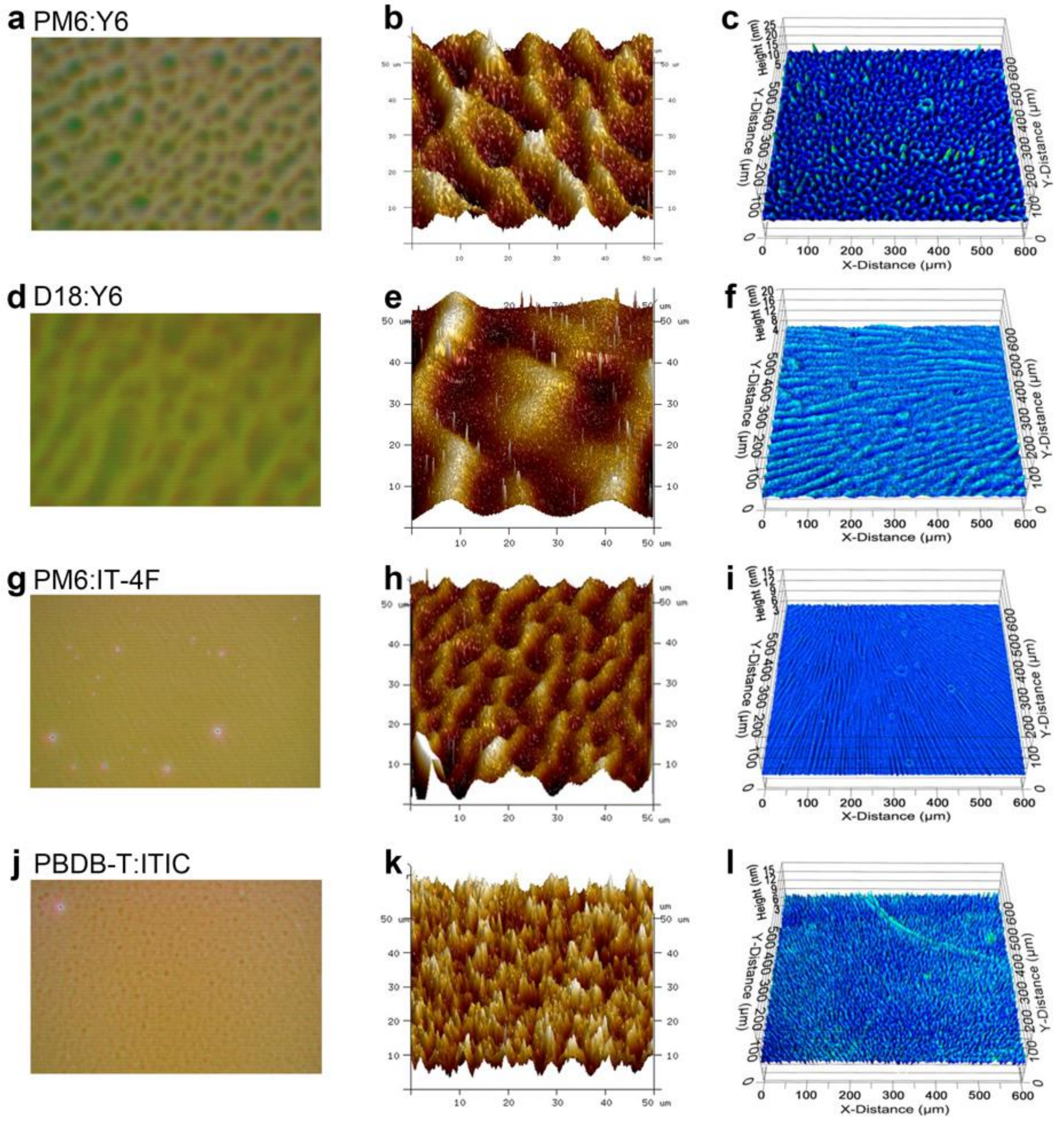

Supplementary Fig. 28 Optical microscope photograph, three-dimensional AFM, surface profile of **a, b, c,** PM6:Y6, **d, e, f,** D18:Y6, **g, h, i,** PM6:IT-4F, **j, k, l,** PBDB-T:ITIC in sequential processing.

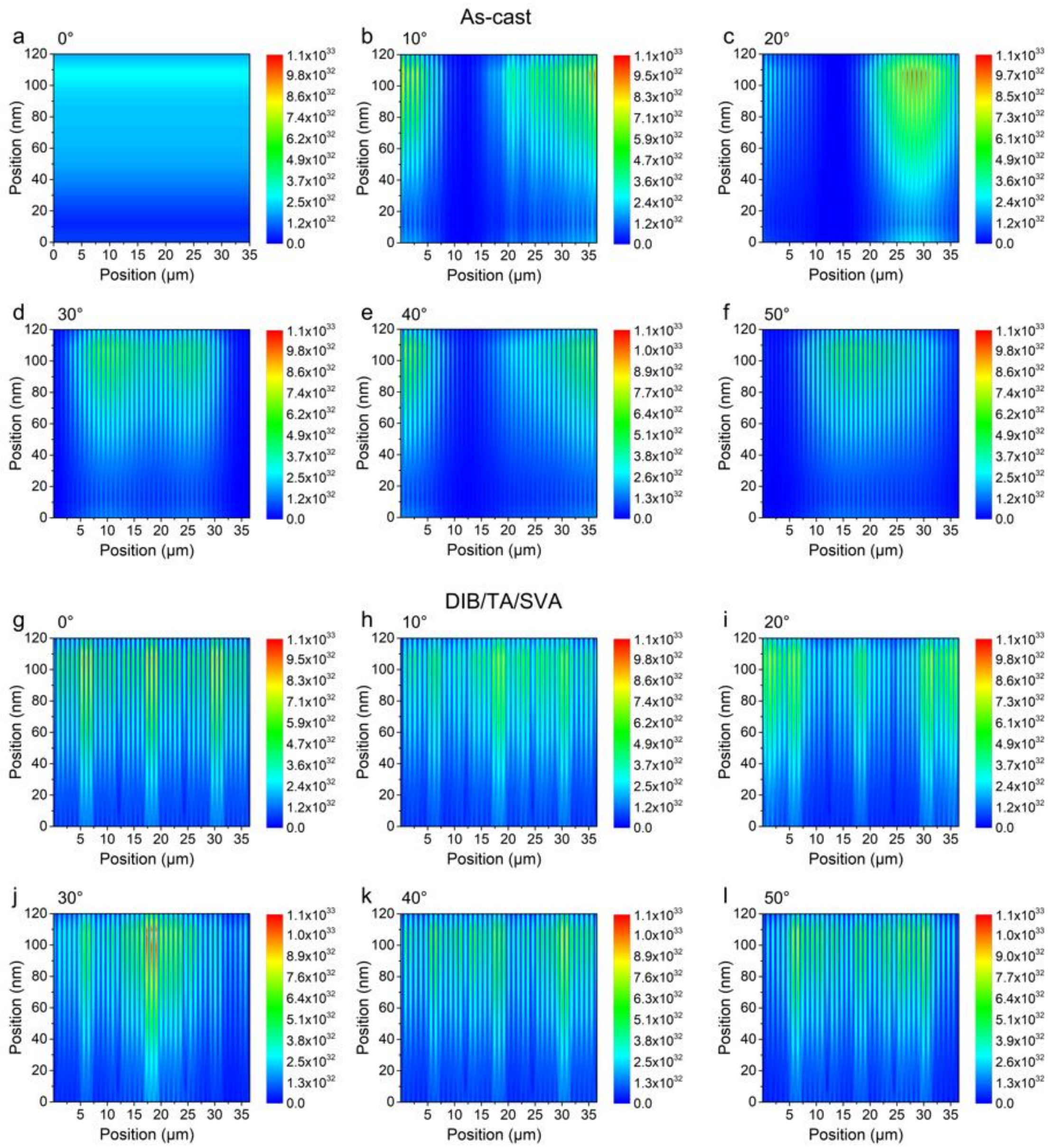

Supplementary Fig. 29 The normalized light field (optical electirc field $|E(x)^2|$) distribution for solar cell devices with different surface morphology at different incident light in **a,** 0° **b,** 10° **c,** 20° **d,** 30° **e,** 40° **f,** 50° (as cast flat device); **g,** 0° **h,** 10° **i,** 20° **j,** 30° **k,** 40° **l,** 50° (DIB/TA/SVA rugged device).

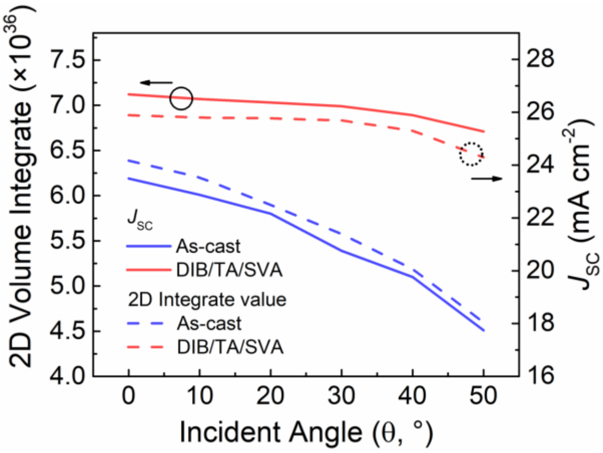

Supplementary Fig. 30 The 2D volume integrate and J_{SC} as function of light incident angle. (The solid blue line and red line represent the J_{SC} for as-cast and DIB/TA/SVA condition at different incident angles, respectively; the dash blue line and red line represent the 2D integrate value of light field distribution for as-cast and DIB/TA/SVA condition at different incident angles, respectively.)

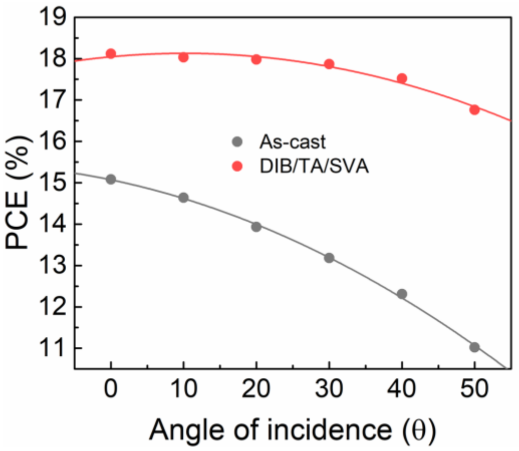

Supplementary Fig. 31 The nonlinear fitting curve of PCE value in different incident angle.

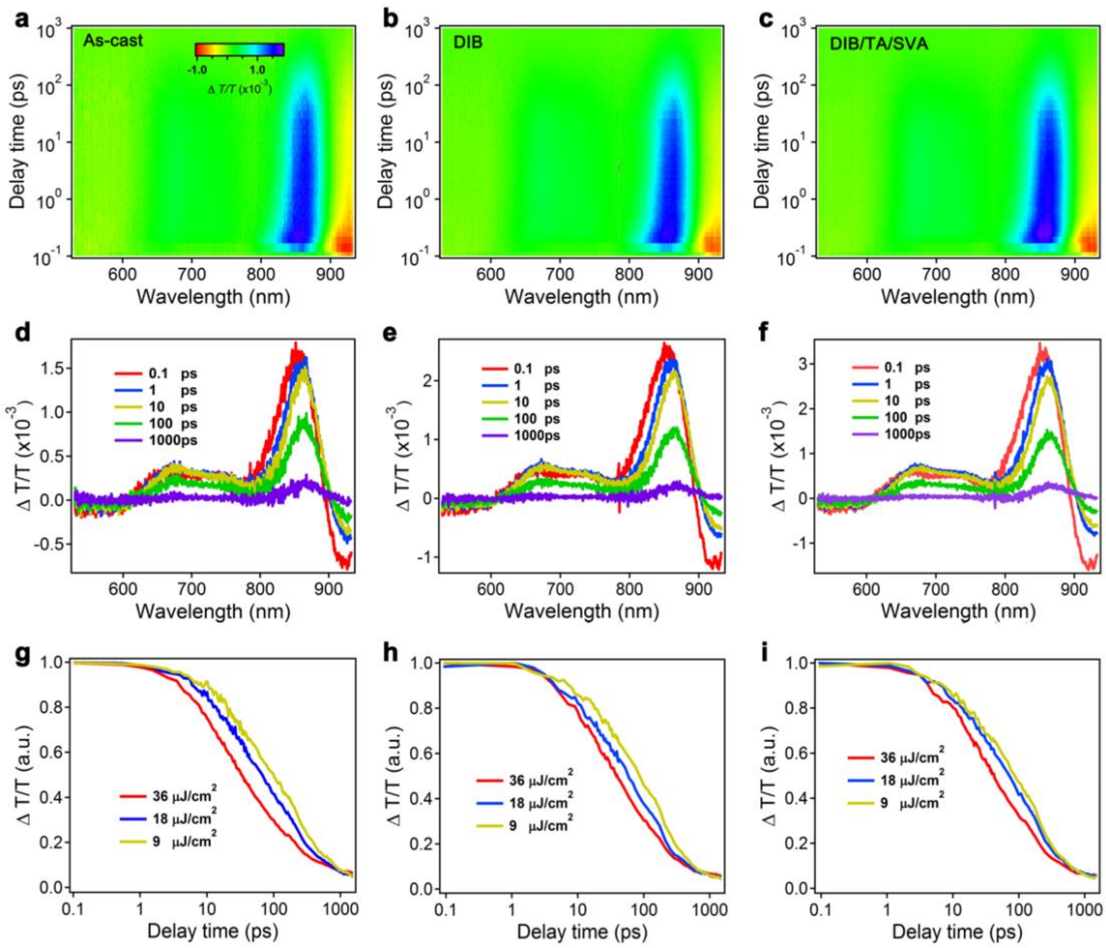

Supplementary Fig. 32 TAS for neat PY-IT films in different conditions. **a-c,** color plot, **d-f,** representative at indicated delay times, **g-i,** the excitons decay dynamics under the excitation of 9, 18 and 36 mJ cm^{-2}.

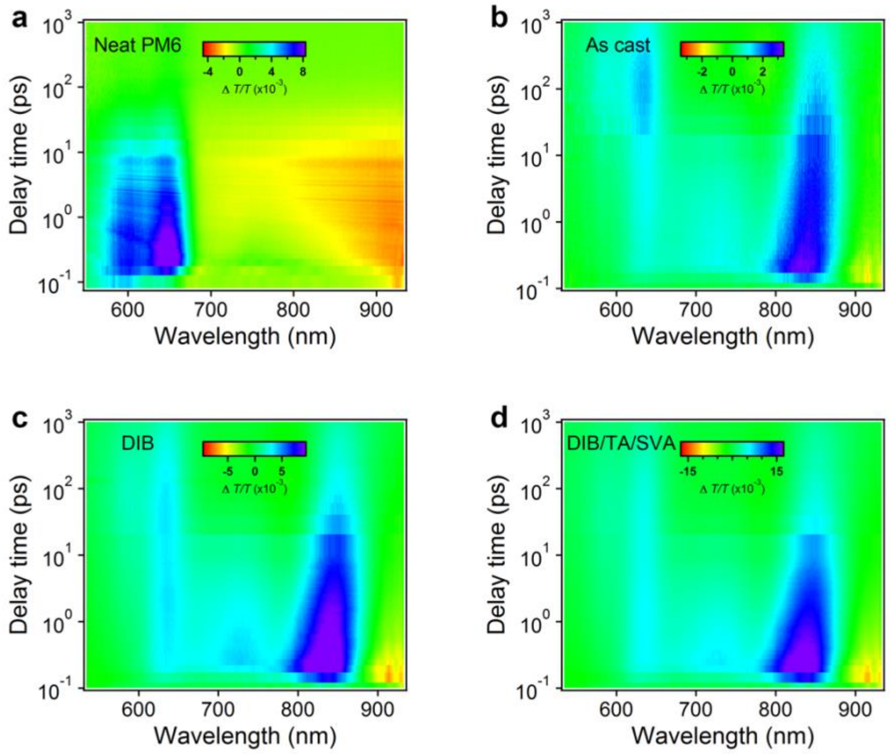

Supplementary Fig. 33 Contour maps of TAS for **a,** neat PM6 (with a color scale from around -4×10^{-3} to 8×10^{-3}), **b,** as-cast blended film (with a color scale from around -3×10^{-3} to 3×10^{-3}), **c,** DIB blended film (with a color scale from around -10×10^{-3} to 10×10^{-3}), and **d,** DIB/TA/SVA blended film (with a color scale from around -15×10^{-3} to 15×10^{-3}).

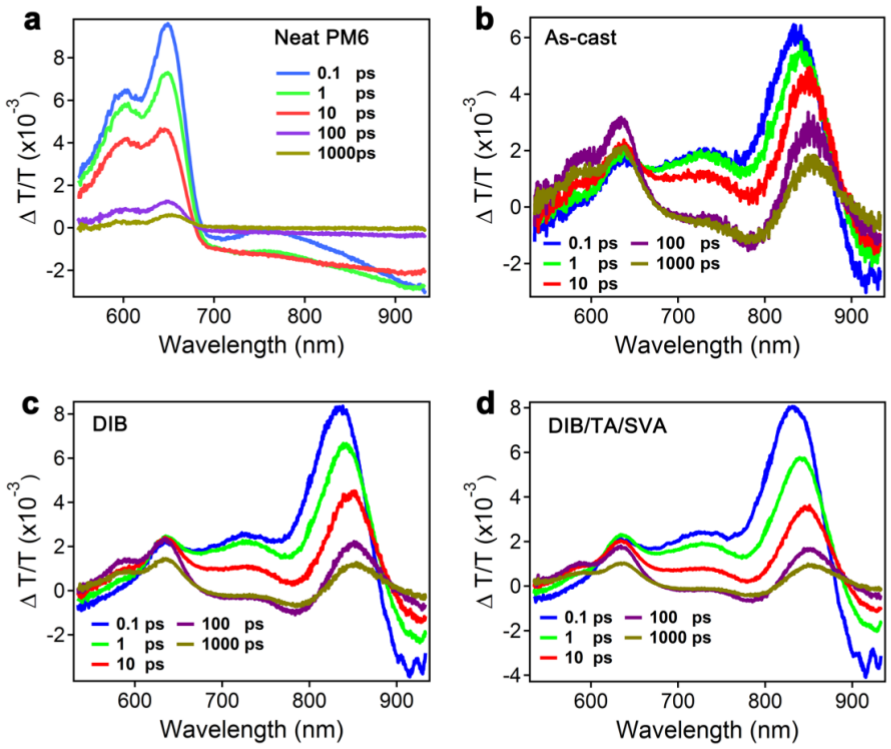

Supplementary Fig. 34 Representative at indicated delay times of TAS for **a,** neat PM6, **b,** as-cast blended film, **c,** DIB blended film, and **d,** DIB/TA/SVA blended film.

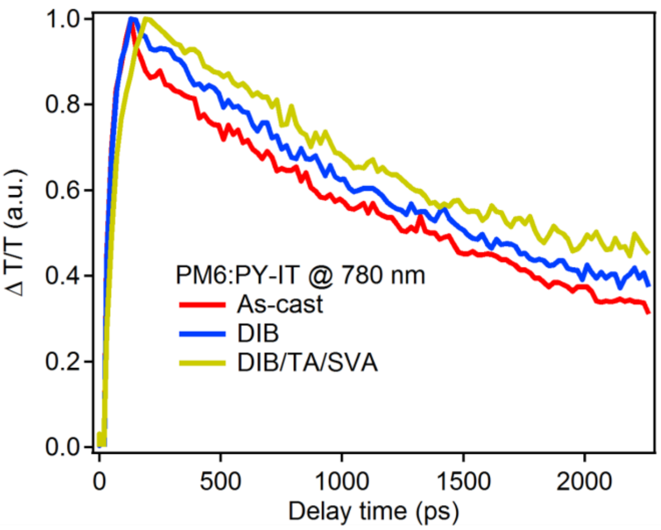

Supplementary Fig. 35 Recombination kinetics of polaron in corresponding conditions.

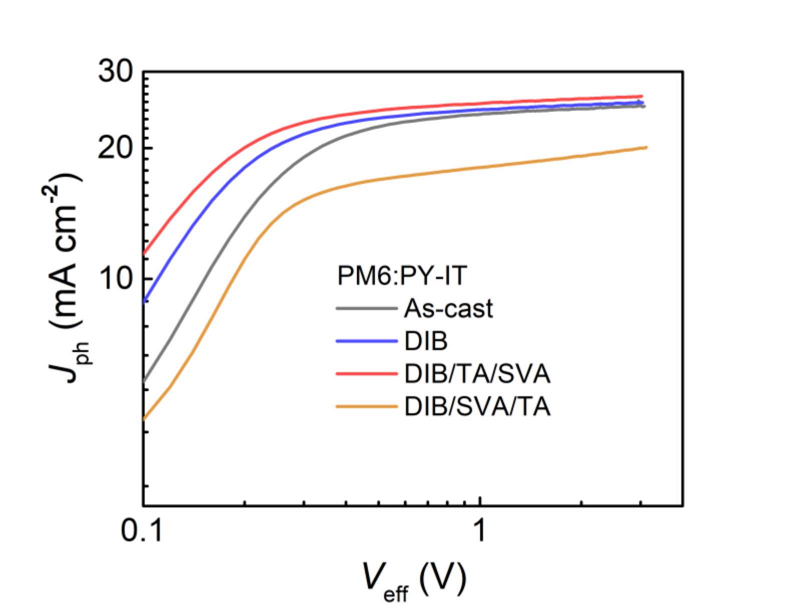

Supplementary Fig. 36 Photocurrent density (J_{ph}) versus effective bias (V_{eff}) characteristics in different conditions.

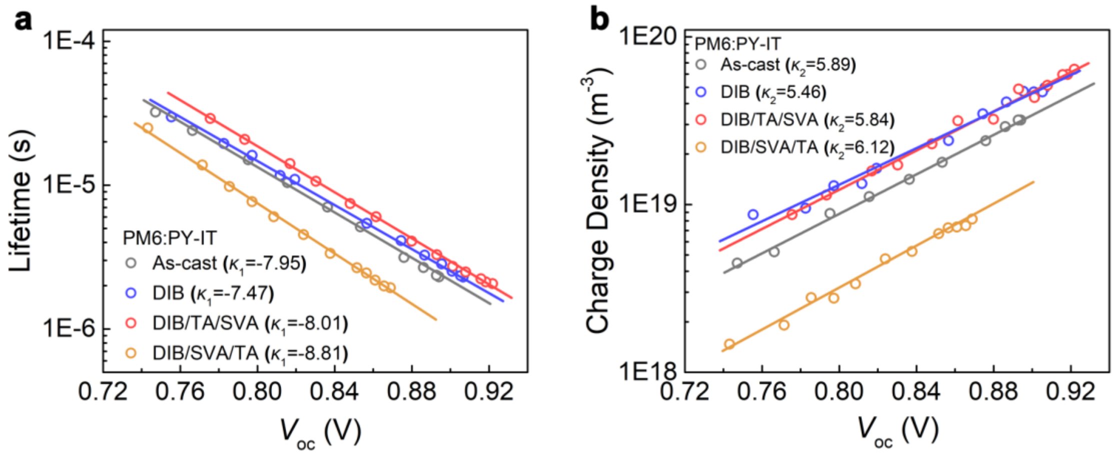

Supplementary Fig. 37 a, Lifetime of carriers and **b,** charge density of carriers as a function of V_{OC} fitted from TPC and TPV. (κ_1 and κ_2 represent the slope of the fitted line for lifetime and charge density as function of V_{OC}, respectively)

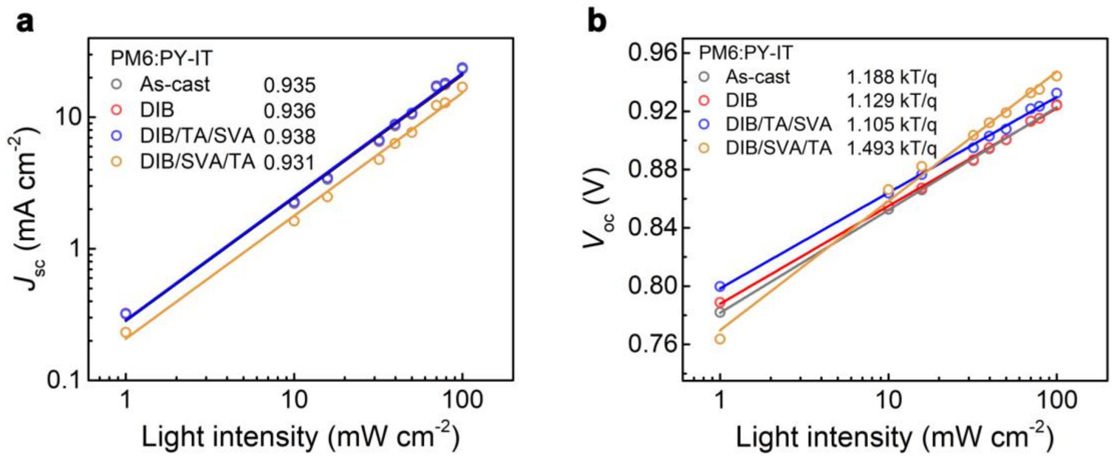

Supplementary Fig. 38 Characteristics of **a,** J_{SC} and **b,** V_{OC} versus light intensity.

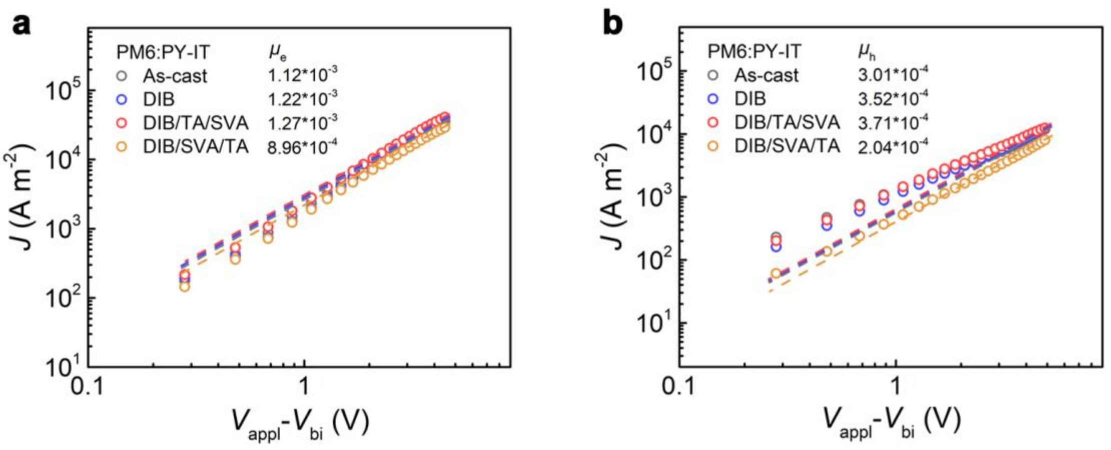

Supplementary Fig. 39 SCLC mobility measurement in different conditions of **a**, electron-only devices and **b**, hole-only devices fitted by Mott-Gurney law. (the μ_e represents electron mobility; the μ_h represents hole mobility)

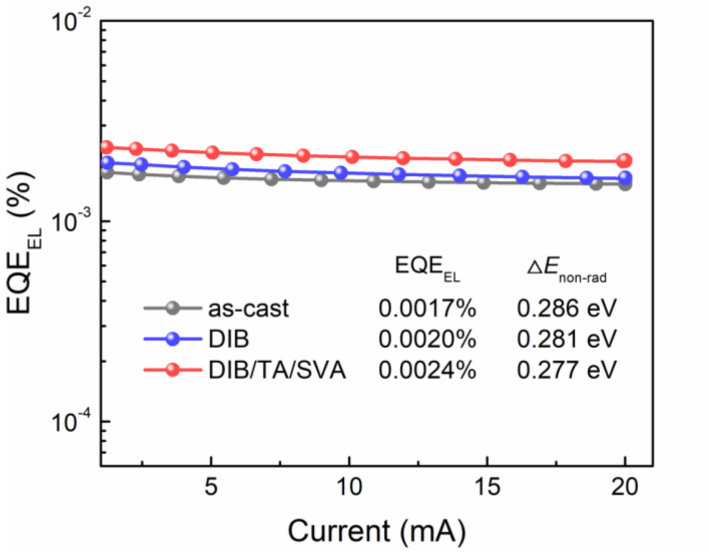

Supplementary Fig. 40 EQE_{EL} of the blend PM6:PY-IT devices. ($\Delta E_{non\text{-}rad}$ represents the non-radiative recombination)

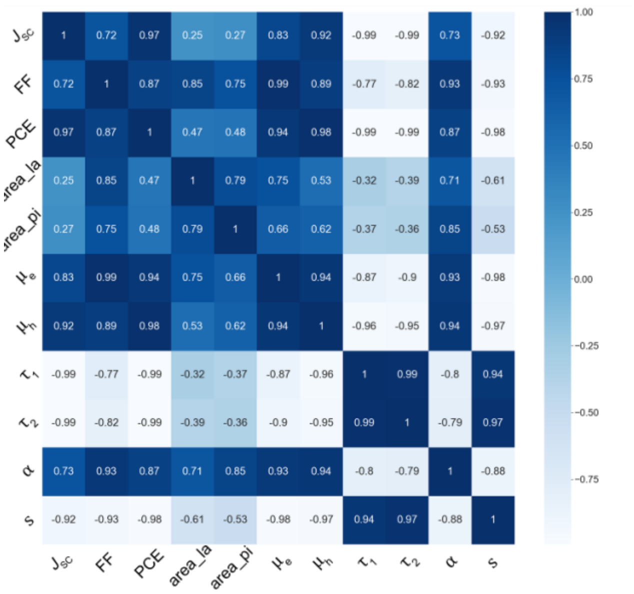

Supplementary Fig. 41 Pearson correlation analysis of major devices (as-cat, DIB, DIB/TA/SVA) and structure parameters.

Supplementary References

1. Ambrosio, A., Devlin, R. C., Capasso, F., & Wilson, W. L. Observation of nanoscale refractive index contrast via photoinduced force microscopy. *ACS Photonics* **4**, 846-851 (2017).
2. Chandrabose, S. et al. High exciton diffusion coefficients in fused ring electron acceptor films. *J. Am. Chem. Soc.* **141**, 6922-6929 (2019).
3. Rivnay, J., Noriega, R., Kline, R. J., Salleo, A., & Toney, M. F. Quantitative analysis of lattice disorder and crystallite size in organic semiconductor thin films. *Phys. Rev. B* **84**, 045203 (2011).
4. Bokel, F. A. et al. In situ X-ray scattering studies of the influence of an additive on the formation of a low-bandgap bulk heterojunction. *Chem. Mater.* **29**, 2283-2293 (2017).
5. Wood, S., Blakesley, J. C., & Castro, F. A. Assessing the validity of transient photovoltage measurements and analysis for organic solar cells. *Phys. Rev. Appl.* **10**, 024038 (2018).
6. Schilinsky, P., Waldauf, C. & Brabec, C. J. Recombination and loss analysis in polythiophene based bulk heterojunction photodetectors. *Appl. Phys. Lett.* **81**, 3885–3887 (2002).
7. Blom, P. W. M., Mihailetchi, V. D., Koster, L. J. A. & Markov, D. E. Device physics of polymer:fullerene bulk heterojunction solar cells. *Adv. Mater.* **19**, 1551–1566 (2007).
8. Fabregat-Santiago, F., Garcia-Belmonte, G., Mora-Sero, I., & Bisquert, J. Characterization of nanostructured hybrid and organic solar cells by impedance spectroscopy. *Phys. Chem. Chem. Phys.* **13**, 9083-9118 (2011).
9. Garcia-Belmonte, G. et al. Influence of the intermediate density-of-states occupancy on open-circuit voltage of bulk heterojunction solar cells with different fullerene acceptors. *J. Phys. Chem. Lett.* **1**, 2566-2571 (2010).
10. Garcia-Belmonte, G., Boix, P. P., Bisquert, J., Sessolo, M., & Bolink, H. J. Simultaneous determination of carrier lifetime and electron density-of-states in

P3HT: PCBM organic solar cells under illumination by impedance spectroscopy. *Sol. Energy Mater. Sol. Cells* **94**, 366-375 (2010).
