# Supplementary material for: All-polymer organic solar cells with nano-to-micron hierarchical morphology and large light receiving angle
Source: Nat Commun. 2023 Jul 12;14:4148. doi: 10.1038/s41467-023-39832-4 (PMC10338490; doi:10.1038/s41467-023-39832-4)
Supplement: Supplementary file 3 — Reporting Summary [file 41467_2023_39832_MOESM3_ESM.pdf]

nature research

Corresponding author(s): Feng Liu

Solar Cells Reporting Summary

Nature Research wishes to improve the reproducibility of the work that we publish. This form is intended for publication with all accepted papers reporting the characterization of photovoltaic devices and provides structure for consistency and transparency in reporting. Some list items might not apply to an individual manuscript, but all fields must be completed for clarity.

For further information on Nature Research policies, including our data availability policy, see Authors & Referees.

Experimental design

Please check: are the following details reported in the manuscript?

1. Dimensions

Item	Yes/No	Details
Area of the tested solar cells	[x] Yes [ ] No	Section "Device Characterization" in the Supplementary Information.
Method used to determine the device area	[ ] Yes [x] No	The area of the device was defined by a shadow mask, which is measured by microscope.

2. Current-voltage characterization

Item	Yes/No	Details
Current density-voltage (J-V) plots in both forward and backward direction	[ ] Yes [x] No	Photo-current hysteresis is not observed in organic solar cells, thus backward direction scan was not applied in this system.
Voltage scan conditions<br>*For instance: scan direction, speed, dwell times*	[x] Yes [ ] No	The current-voltage curves were measured along the reverse scan direction from -0.2 to 1 V, yielding identical results. The scan speed and dwell times were fixed at 0.05 V/step and 10 ms, respectively. (Section "Device Characterization" in the Supplementary Information)
Test environment<br>*For instance: characterization temperature, in air or in glove box*	[x] Yes [ ] No	The current-voltage curves were measured in a glove box at room temperature. (Section "Device Characterization" in the Supplementary Information)
Protocol for preconditioning of the device before its characterization	[ ] Yes [x] No	No preconditioning protocol.
Stability of the J-V characteristic<br>*Verified with time evolution of the maximum power point or with the photocurrent at maximum power point; see ref. 7 for details.*	[x] Yes [ ] No	The stability performance were shown in Supplementary Fig. 11.

3. Hysteresis or any other unusual behaviour

Item	Yes/No	Details
Description of the unusual behaviour observed during the characterization	[ ] Yes [x] No	No hysteresis or other unusual behavior was observed during the characterization of the solar cells. In general, organic solar cells do not have hysteresis problems.
Related experimental data	[ ] Yes [x] No	No hysteresis or other unusual behavior was observed during the characterization of the solar cells.

4. Efficiency

Item	Yes/No	Details
External quantum efficiency (EQE) or incident photons to current efficiency (IPCE)	[x] Yes [ ] No	As shown in Fig. 1c.
A comparison between the integrated response under the standard reference spectrum and the response measure under the simulator	[ ] Yes [x] No	We have reported related data in former paper.
For tandem solar cells, the bias illumination and bias voltage used for each subcell	[ ] Yes [x] No	We only fabricated single-junction solar cells.

5. Calibration

Item	Yes/No	Details
Light source and reference cell or sensor used for the characterization	[x] Yes [ ] No	Section "Device Characterization" in the Supplementary Information.

Confirmation that the reference cell was calibrated and certified

☒ Yes ☐ No

Section "Device Characterization" in the Supplementary Information.

Calculation of spectral mismatch between the reference cell and the devices under test

☒ Yes ☐ No

Silicon cells were used to rectify before JV curve and EQE measurement.

6. Mask/aperture

Size of the mask/aperture used during testing

☒ Yes ☐ No

All samples were measured with a 0.0313 cm^2 mask for cells.

Variation of the measured short-circuit current density with the mask/aperture area

☐ Yes ☒ No

All the current-voltage curves were measured with a single mask.

7. Performance certification

Identity of the independent certification laboratory that confirmed the photovoltaic performance

☒ Yes ☐ No

A recorded efficiency of 18.59% was certified by Chengdu Institute of Product Quality Inspection Co., Ltd. National Photovoltaic Product Quality Inspection &Testing Center.

A copy of any certificate(s)
Provide in Supplementary Information

☒ Yes ☐ No

See "Supplementary Fig. 10" in the Supplementary Information.

8. Statistics

Number of solar cells tested

☒ Yes ☐ No

The device performance statistics was based on around 30 devices.

Statistical analysis of the device performance

☒ Yes ☐ No

Results were included in Fig. 1d,e.

9. Long-term stability analysis

Type of analysis, bias conditions and environmental conditions
For instance: illumination type, temperature, atmosphere humidity, encapsulation method, preconditioning temperature

☒ Yes ☐ No

Supplementary Fig. 11 shows the normalized performance of encapsulated device by UV adhesive package under continuous illumination over 1200h under white light illumination of 1 sun intensity in air at room temperature.
